# Changes in Parasitoid Communities Over Time and Space: A Historical Case Study of the Maize Pest *Ostrinia nubilalis*

**DOI:** 10.1371/journal.pone.0025374

**Published:** 2011-09-30

**Authors:** Laurent Folcher, Denis Bourguet, Denis Thiéry, Laurent Pélozuelo, Michel Phalip, Alain Weissenberger, Nathalie Eychenne, Catherine Regnault-Roger, Marc Delos

**Affiliations:** 1 Université de Pau et des Pays de l'Adour, Unité Mixte de Recherche 5254, Institut Pluridisciplinaire pour l'Environnement et les Matériaux, Equipe Environnement et Microbiologie, Centre National de la Recherche Scientifique, Pau, France; 2 Centre de Biologie pour la Gestion des Populations, Unité Mixte de Recherche, Institut National de la Recherche Agronomique, Montferrier-sur-Lez, France; 3 Institut des Sciences de la Vigne et du Vin, Unité Mixte de Recherche 1065 Santé et Agroécologie du Vignoble, Institut National de la Recherche Agronomique, Villenave d'Ornon, France; 4 Université de Toulouse, Laboratoire d'Ecologie Fonctionnelle et Environnement, Unité Mixte de Recherche 5245, Toulouse, France; 5 Service Régional de l'Alimentation, Direction Régionale de l'Agriculture et de la Forêt “Poitou Charentes”, Biard, France; 6 Chambre d'Agriculture du Bas-Rhin, Schiltigheim, Strasbourg, France; 7 Fédération Régionale de Défense contre les Organismes Nuisibles “Midi-Pyrénées”, Castanet Tolosan, France; 8 Service Régional de l'Alimentation, Direction Régionale de l'Agriculture et de la Forêt “Midi-Pyrénées”, Toulouse, France; University of Western Ontario, Canada

## Abstract

Understanding the ways in which human environmental modifications affect biodiversity is a key challenge in conservation planning, pest control and evolutionary ecology. Parasitoid communities, particularly those associated with agricultural pests, may be susceptible to such modifications. We document here changes in the larval parasitoid communities of *Ostrinia nubilalis* — the main pest of maize — and its sibling species *O. scapulalis*, based on two historical datasets, one collected from 1921–1928 and the other from 2001–2005. Each of these datasets encompasses several years and large geographical areas and was based on several thousands/millions of host larvae. The 80-year interval between the two datasets was marked by a decrease in *O. nubilalis* parasitism to about two thirds its initial level, mostly due to a decrease in the rate of parasitism by hymenopterans. However, a well balanced loss and gain of species ensured that species richness remained stable. Conversely, *O. scapulalis* displayed stable rates of parasitism over this period, with a decline in the species richness of its parasitoid community. Rates of parasitism and species richness in regions colonized by *O. nubilalis* during the 1950s were one half to one third those in regions displaying long-term colonisation by this pest. During the recent human activity-driven expansion of its range, *O. nubilalis* has neither captured native parasitoids nor triggered parasite spill back or spill over.

## Introduction

Human activity exerts strong selective pressures on all kind of species and on communities of species [1]–[6]. These selective pressures arise from diverse sources, including global warming, the use of pesticides and drugs in agriculture and medicine, land transformation (land clearing, fire suppression, cultivation, deforestation, urbanization etc.) and pollutants from industry and various types of traffic. One of the key challenges in conservation planning, pest control and evolutionary ecology in general is understanding the ways in which such changes affect biodiversity and community structures [7], [8]. For example, trade and human travel may promote biodiversity by favouring the introduction of alien species at a rate exceeding the rate of replacement of native species [9]. Landscape fragmentation can increase the heterogeneity of the environment, resulting in a diversity of habitats favouring a high species richness [10], [11]. The recent climate changes associated with global warming have also allowed many species to expand their ranges [12], [3]. However, in most cases, habitat fragmentation and environmental degradation lead to a decrease in species richness in most communities of species [13]–[16].

Urbanization and environmental degradation have been shown to have detrimental effects on species abundance, species richness and eveness in diverse groups of insects [17]–[24], including parasitoid communities [10], [18], [25]–[28]. These insects, like all natural enemies of arthropods, are intrinsically susceptible to changes due to human activity, because they are influenced by both the environmental variations themselves and by the unpredictable changes in host dynamics induced by these variations. Parasitoid communities have been shown to be affected by habitat type [29], [30], and to be more sensitive to habitat fragmentation than their phytophagous hosts [31]–[33].

Most studies of the changes in parasitoid communities induced by human activity have compared species richness, abundance and diversity at various geographical sites or over geographical gradients in climatic conditions (e.g., [34]), level of urbanization (e.g., [27], [35]), landscape structure (e.g., [25]) or soil fertility (e.g., [36]). Studies of changes over time have tended to focus on a restricted period of several years, with few studies dealing with periods of more than five to 10 years (e.g., [37]). However, efforts to assess the influence of human activity on arthropod communities would benefit greatly from longitudinal surveys of the demographics of phytophagous insects and their associated parasitoids [34]. Unfortunately, no such comparisons of communities of species over longer time periods have been published. This is at least partly because there are far fewer historical datasets for insects than for vertebrates, such as birds in particular [38].

In the particular case of pest species, parasitoid communities must not only contend with the “classical” challenges of global warming, pesticide treatments, crop harvest and tillage, they must also cope with the extension or regression of their host's geographical range. Variations in the communities of parasitoids attacking agricultural pests are ultimately linked to changes in the cultivation of the crop on which these pests feed. According to the enemy release hypothesis, species with an expanding range can escape their parasites and therefore suffer a lower parasite burden, which in turn facilitates the expansion of their range [39]–[41]. Parasitoids following their hosts into new areas may eventually parasitise native hosts in the newly colonized habitats, a situation known as parasite “spill over” [42], [43]. Finally, parasitoids already established in areas into which the species with the expanding range moves may switch host to the invader from resident host species related to the invader, triggering a parasite “spill back' process [42], [44], [45]. Enemy release, parasite “spill over” and “spill back” have been largely explored for alien host species in the framework of biological invasion [41]. Conversely, only a few empirical studies have explored the extent to which expansion of the range of a species affects or is affected by the new communities encountered (but see [46]–[50]).

For historical, political and economic reasons, the European corn borer (ECB), *Ostrinia nubilalis* Hübner (Lepidoptera: Crambidae), provides a unique opportunity to investigate changes in a parasitoid community after a significant period of time marked by profound changes due to human activity and differences in that community between areas of recent or long-established infestation. This moth is currently the main pest of maize, *Zea mays* L., throughout the world. It is native to Eurasia and was accidentally introduced into the United States about a hundred years ago [51]. From 1920 to 1937, possible control of this pest by biological agents, including parasites, predators and diseases, was considered in detail by the United States Department of Agriculture (USDA). Over this 20-year period, more than 23 million *Ostrinia* larvae from Europe and 3 million larvae from the Far East were collected and brought to the United States, and the natural enemies they contained were reared and then released into the fields [52]. Much of the European sampling took place in France and Thompson & Parker [53] and Parker et al. [54] provided a detailed summary of the rates of parasitism of ECB larvae collected in France from 1921 to 1928. At the time, *O. nubilalis* was considered a highly polyphagous species. Sampling was therefore carried out not only on maize, in southern France where this crop was widely grown, but also on mugwort, *Artemisia vulgaris* L., in the northern and western parts of France in which maize was not grown [53], [55]. Meanwhile, population genetics and ecological studies showed that the *Ostrinia* larvae feeding on mugwort actually belonged to *O. scapulalis*, a sibling species of *O. nubilalis* [56]. Although interfertile and similar morphologically, these two species are genetically differentiated from each other and feed on different host plants: mugwort, hop (*Humulus lupulus* L.) and hemp (*Cannabis sativa* L.) for *O. scapulalis* and maize for *O. nubilalis* (see [56] for a review). For unknown reasons, *O. scapulalis* is mostly restricted to northern France [53], [57]. A few larvae of this species have been detected on mugwort in central France [53], [54] but it does not infest this weed in southern France [53], [57]. The dataset from the USDA therefore provides a precise snapshot of the parasitoid communities infesting two sibling host species in the 1920s, a time at which these two species were largely allopatric.

At the start of the 21^st^ century, the French Ministry of Agriculture launched a five-year programme (2001–2005) with the aim of characterising the parasitoid community infesting ECB larvae throughout France before the potential introduction of Bt maize varieties, which produce Cry toxins active against ECB larvae. This dataset was described in part by Folcher et al. [58] and Pélissié et al. [59], who also provided information about the parasitoid community infesting *O. scapulalis* larvae on mugwort during the 2000s. This second massive sampling campaign provided a description of the parasitoid communities infesting *O. nubilalis* and *O. scapulalis* larvae some 80 years after the USDA campaign. During the interval between these two sampling campaigns, in the 1950s, maize cultivation was expanded in France, and *O. nubilalis* became sympatric, in western and northern France, with *O. scapulalis* feeding on mugwort.

We provide here an analysis of the complete dataset for the larval ECB parasitoid community obtained from 2001 to 2005 by the French Ministry of Agriculture. We compared this dataset with the USDA dataset for 1921–1928, to determine (i) whether and to what extent the larval parasitoid communities of *O. nubilalis* and *O. scapulalis* had changed over a 80-year period marked by intense environmental modifications, (ii) whether these changes were of similar magnitude for the two main groups of parasitoids, tachinids and hymenopterans and (iii) whether the expansion of *O. nubilalis* into the range of *O. scapulalis* driven by human activity was accompanied by changes in diversity and, more specifically, by any phenomenon of parasite release, “spill over” or “spill back”.

## Results

During the 2001 to 2005 sampling campaign, 1,307 tachinid flies and 423 hymenopteran wasps emerged from the 42,688 ECB larvae collected on maize over all the sites and all the years considered. The different species recovered are listed in Table 1. The correspondence with the taxonomic nomemclature used by the USDA teams during the 1920's is given in Table S1. For each species, the mean *PR* per site for each region and for each of the five years is provided in the supporting information, in Tables S2 and S3. Our stepwise GLM analysis of this dataset showed that, with only one exception, the factor “year” and the interactions “year x region” and “year x group of regions” did not influence *PR*, *SR*, *SP* and *H′* for tachinids, for hymenopterans or all parasitoids considered together (see supporting information, Tables S4 and S5 for details on *F* and χ^2^ values, degree of freedom and *p*-values). Conversely, we found significant differences in *PR*, *SR*, *SP* and *H′* between regions or groups of regions for tachinids, hymenopterans and all parasitoids (supporting information, Tables S4 and S5).

**Table 1:** Parasitoid species identified within communities infesting *O. nubilalis* and *O. scapulalis*.

		*O. nubilalis*		*O. scapulalis*
Family	Species	1921–1928	2001–2005	1921–1928
Tachinidae	*Actia pilipennis* Fallen, 1810		+	
	*Lydella thompsoni* Herting, 1959	+	+	+
	*Nemorilla maculosa* Meigen, 1824	+		
	*Pseudoperichaeta nigrolineata* Walker, 1853	+	+	
	*Pseudoperichaeta palesoidea* Robineau-Desvoidy, 1830		+	
	*Voria ruralis* Fallen, 1810		+	
	*Zenillia mitis* Meigen, 1824			+
Braconidae	*Apanteles thompsoni* Lyle, 1927	+		+
	*Macrocentrus cingulum* Brischke, 1882			+
	*Bracon brevicornis* Wesmael, 1838	+	+	+
	*Microgaster messoria* Haliday, 1834	+	+	+
Eulophidae	Eulophid sp.			+
	Eulophus sp.	+		
Ichneumonidae	*Campoplex lugubrinus* Holmgren, 1855			+
	*Campoplex rothi* Holmgren, 1855			+
	*Diadegma fenestrale* Holmgren, 1860		+	
	*Eriborus terebrans* Gravenhorst, 1829	+	+	+
	*Exeristes roborator* Fabricius, 1793	+		+
	*Phaogenes planifrons* Wesmael, 1877	+		+
	*Pristomerus vulnerator* Panzer, 1799		+	
	*Sinophorus turionum* Ratzeburg, 1844	+	+	+
	*Theronia atalantae* Poda, 1761	+		

As *PR*, *SR*, *SP* and *H′* were stable from year to year in a given region or group of regions in the 2001–2005 period, these rates were formally comparable over space, between *ancestral*, *intermediate* and *newly colonized* regions. The *PR* recorded by USDA teams in 1921 to 1928 – given per region in Tables S6, S7, S8, S9 – were also stable over time [53], [54], making it possible investigate the long-term changes over time in the larval parasitoid communities infesting *O. nubilalis* and *O. scapulalis*.

### Changes in the parasitoid community infesting *Ostrinia* over time

**Figure 1:**
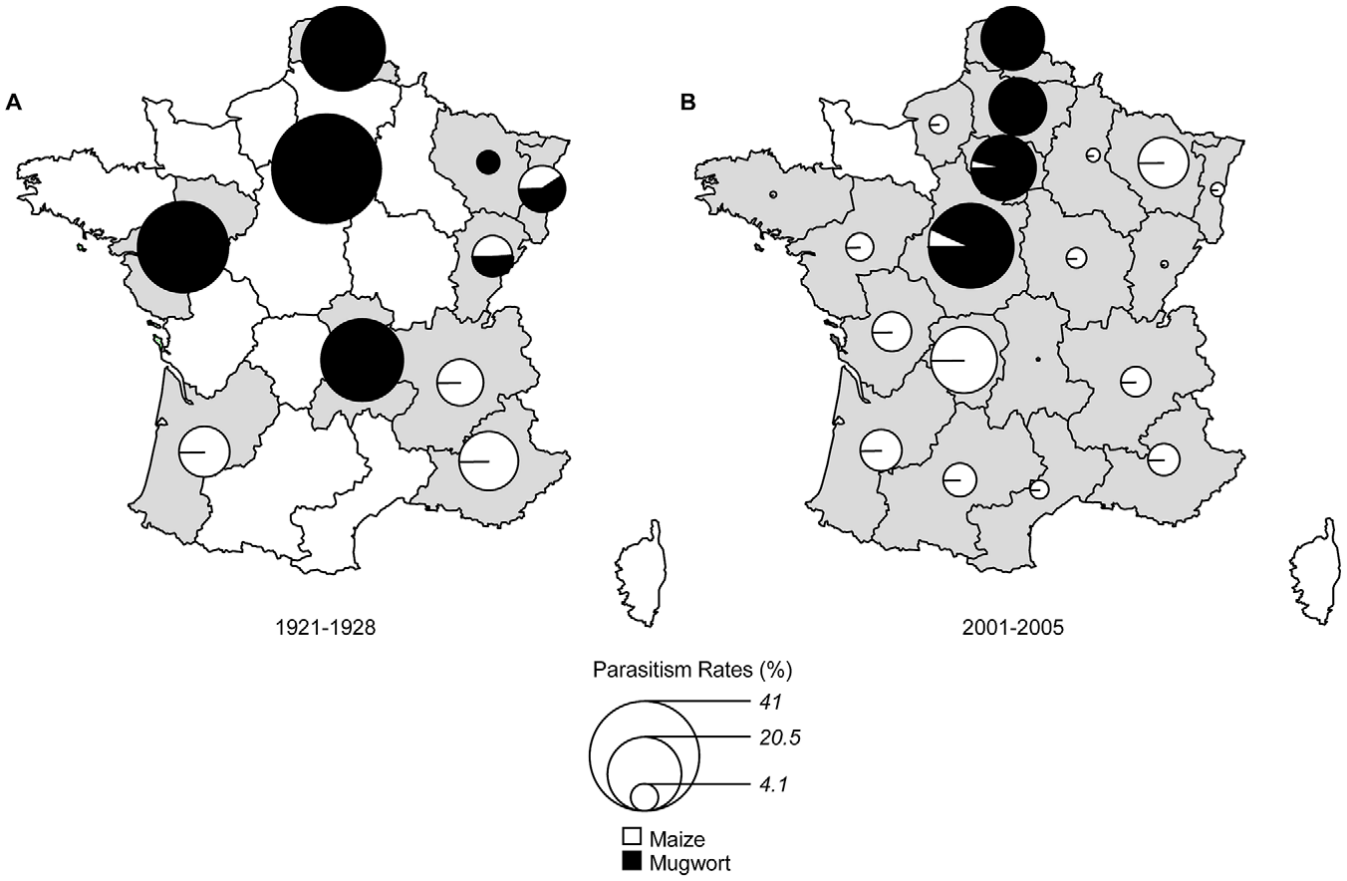
Mean parasitism rate per site for all parasitoids infesting *O. nubilalis* feeding on maize (period 1921–1928 (*A*) and 2001–2005 (*B*)) and *O. scapulalis* on mugwort (period 1921–1928 (*C*) and 2001–2005 (*D*)).

In the five regions for which comparisons were possible, the overall *PR* of ECB by parasitoids was lower in 2001–2005 than in 1921–1928 (Figures 1A and 1B, Table 2). *PR* was found to have decreased by a factor of 1.3 to 18, but this decrease was significant only for Provence-Alpes-Côte-d'Azur (*t_7_* = 2.79, *p* = 0.027), probably due to the low statistical power. The *PR* over all regions significantly (*t_35_* = 4.43, *p*<0.001) decreased, from 11.2% to 4.0% (Table 2). The last 80 years of the 20^th^ century were therefore marked by a two thirds decrease in parasitoid levels.

**Table 2:** Temporal comparison of *O. nubilalis* parasitoid communities in all regions sampled by the USDA in 1921–1928.

				Mean *PR* per site						
Region	Period	*n* sites	*n* larvae	Tachinids	Hymenopterans	Total parasitoids	*SR*	Mean *H′*	*t*-value	*p*-value
Alsace	1921–1928	*	500	0.00	7.80	7.80	2	0.24	-	-
	2001–2005	28	5,483	0.76±1.19	0.42±0.73	1.22±1.42	5	0.05±0.05		
Aquitaine	1921–1928	*	**	6.18±4.33	4.61±4.04	10.79±5.59	8	0.33±0.12	1.03(*df* = 7)	0.339
	2001–2005	25	3,886	6.14±1.90	0.92±0.37	7.75±2.09	10	0.26±0.05		
Franche-Comté	1921–1928	*	>1,250	0.45	9.28±5.16	8.10	4	0.19	-	-
	2001–2005	9	969	0.00	0.35±0.40	0.44±0.54	1	-		
Provence-Alpes-Côte d'Azur	1921–1928	*	>3,333	10.34±5.06	5.30±5.74	15.08±15.25	10	0.41±0.13	2.79(*df* = 7)	0.027
	2001–2005	5	886	2.71±2.41	1.37±1.48	4. 99±3. 69	3	0.18±0.04		
Rhône-Alpes	1921–1928	*	**	6.85±3.89	4.74±4.18	9. 39±6.78	6	0.34±0.21	1.32(*df* = 7)	0.228
	2001–2005	30	5,293	1.83±1.75	1.83±1.41	4.43±2.48	7	0.15±0.09		
All regions	1921–1928	*	**	6.71±4.94	5.89±4.51	11. 21±5.61	12	0.34±0.15	4.43(*df* = 35)	<0.001
	2001–2005	97	16,517	2.49±2.68	1.03±1.11	4.05±3.42	11	0.17±0.09		

The indices calculated were parasitism rates (*PR*, mean % ± s.d.), species richness (*SR*), Shannon and Weaver's diversity index (*H′*, mean bits ± s.d.),

* = not available, but probably many tens (53),

**not given, but probably several thousand (53).

**Figure 2:**
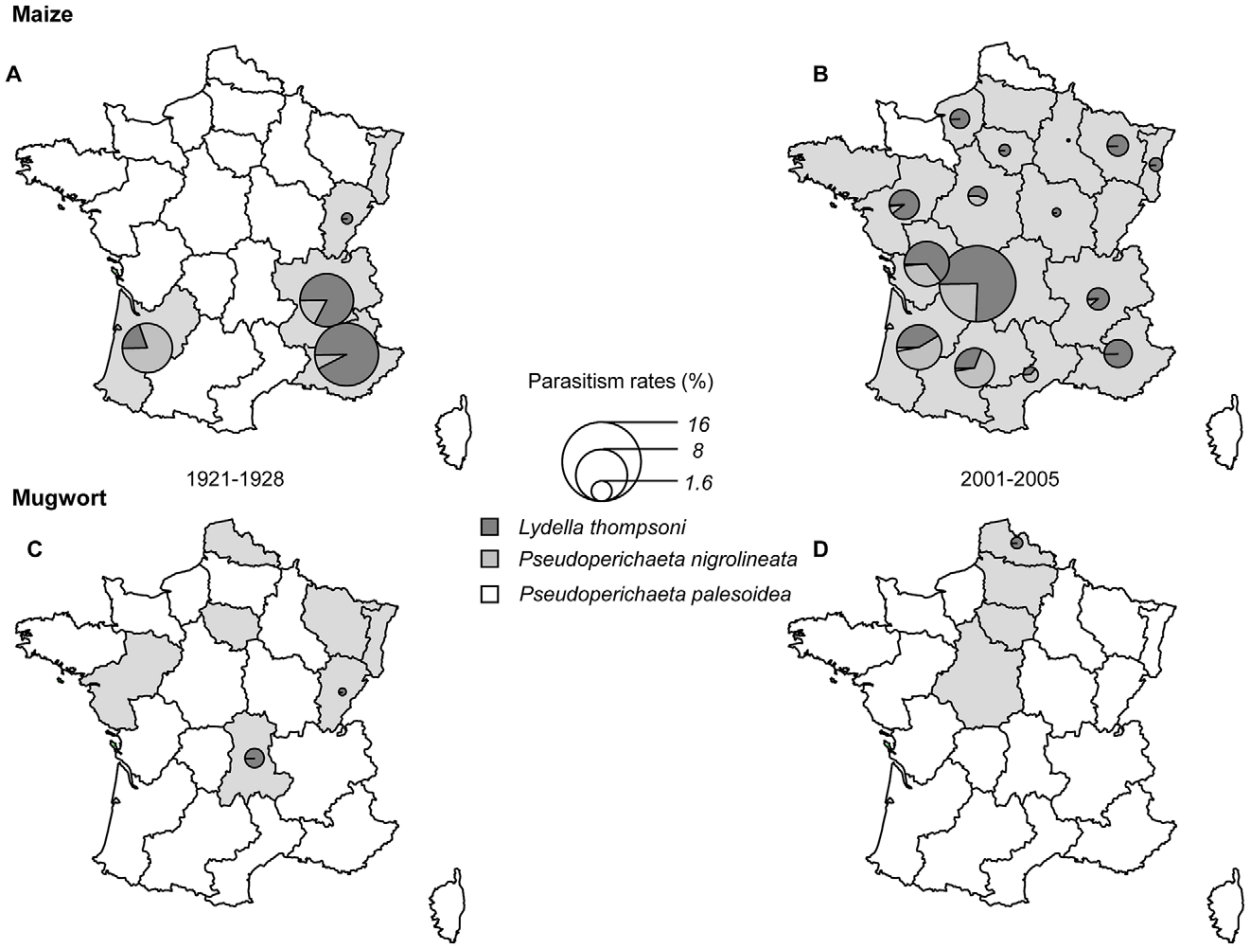
Mean parasitism rate per site for the three main tachinid species infesting *O. nubilalis* feeding on maize (period 1921–1928 (*A*) and 2001–2005 (*B*)) and *O. scapulalis* on mugwort (period 1921–1928 (*C*) and 2001–2005 (*D*)).

This change in *PR* was driven mostly by changes in the hymenopteran community. In the 2001–2005 period, the abundance of hymenopterans was only 17% that in the 1921–1928 period (i.e., 1.03 *vs* 5.89%). The decrease in tachinid levels was also significant, but to a lesser extent. The abundance of these parasitoid flies decreased by about 60% over all regions (i.e., 2.49 *vs* 6.71%) but remained extremely stable over the two periods of time in Aquitaine (Figures 2A and 2B, Table 2).

**Figure 3:**
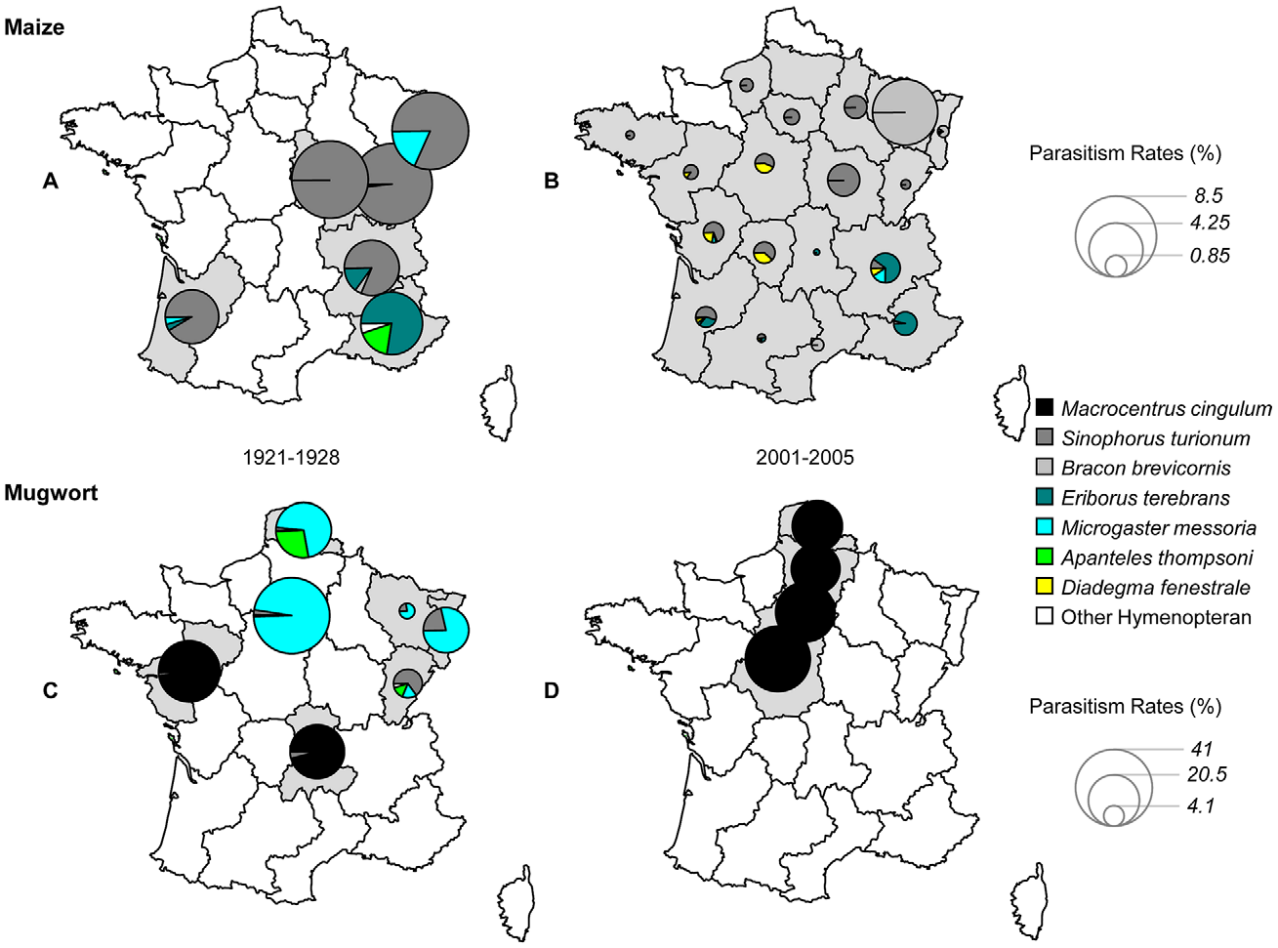
Mean parasitism rate per site for hymenopteran species infesting *O. nubilalis* feeding on maize (period 1921–1928 (*A*) and 2001–2005 (*B*)) and *O. scapulalis* on mugwort (period 1921–1928 (*C*) and 2001–2005 (*D*)).

A decrease in *H′* between the two periods of time was also noted in all *ancestral* regions (Table 2). This difference in *H′* resulted mostly from the decrease in *PR* over time documented above. Indeed, *SR* remained similar for the five regions considered, with 12 species recorded during the 1920s and 11 species recorded during the 2000s (Table 2), despite significant changes in the composition of the parasitoid communities. *L. thompsoni* and *P. nigrolineata* were the two principal tachinid fly species infesting *O. nubilalis*, in both 1921–1928 and 2001–2005 (Table 1). However, *P. palesoidea*, a species not present during the 1920s, was recorded in Aquitaine and Rhône-Alpes during the sampling campaigns in the 2000s (Figure 2B). Among hymenopterans, *A. thompsoni* was no longer recorded in 2001–2005 and parasitism by *M. messoria* became much rarer than reported in 1921 to 1928 (Figures 3A and 3B). Conversely, the 2001–2005 period was characterised by the emergence of *D. fenestrale* (Figure 3B), an ichneumonid never recorded during the 1920s. Finally, *S. turionum* was the only hymenopteran species found at relatively similar frequencies in ECB populations in both time periods.

Pélissié et al. [59] provided *PR* values for *O. scapulalis* collected on mugwort in four regions of France in 2002. These *PR* values (20–40%) were similar to those recorded during the USDA campaigns in 1921 to 1928 (Figure 1B). Pélissié et al. [59] also found that *Macrocentrus cingulum* was the only hymenopteran parasitoid emerging from those diapausing larvae at all sites (Figures 2D and 3D). Similar results – i.e., *PR*, varying from 20 to 40%, exclusively due to *M. cingulum* – were obtained in subsequent samplings performed in 2003, 2005, 2009 and 2011 (details not shown). *M. cingulum* was the predominant or only parasitoid infesting *O. scapulalis* in only two of the seven regions in the 1920s (Figures 2C and 3C). In the other five regions, this parasitoid was either absent or present at a very low frequency (Figure 3C). The three other main hymenopteran species recorded in these five regions, *A. thompsoni*, *M. messoria* and *S. turionum*, were not found by Pélissié et al. [59] in the samples collected in 2002. The absence of both *M. messoria* and *S. turionum* could be accounted for by the biological features of these two species. Indeed, these two species emerge from *Ostrinia* larvae during the autumn and diapause in their own cocoon [53]. In 2002, diapausing larvae of *O. scapulalis* were collected during the winter, and were therefore necessarily free of both *M. messoria* and *S. turionum*. The absence of *A. thompsoni* cannot be explained so easily, because it has a life cycle very similar to that of *M. cingulum*, diapausing within the *Ostrinia* larvae [53].

### Changes in the parasitoid community during expansion of the range of *O. nubilalis*

The mean *PR* per site in the *newly colonized* regions was half that in the *ancestral* regions (Table 3), and this difference was highly significant (Tukey's HSD test, *p*<0.001). This halving of the *PR* value concerned both tachinids and hymenopterans. However, the mean *PR* per site in the *intermediate* regions was twice that in *ancestral* regions and four times that in *newly colonized* regions, (Table 3); these differences were highly significant (Tukey's HSD test, *p* = 0.005 and *p*<0.001, respectively). Both tachinids and hymenopterans had these higher *PR* values in the *intermediate* regions (Table 3).

**Table 3:** Comparison of the biodiversity of *O. nubilalis* parasitoid communities between *ancestral*, *intermediate* regions and regions *newly colonized* with ECB.

	Tachinids			Hymenopterans			Total parasitoids		
	*Ancestral*regions	*Intermediate*regions	*Newly colonized*regions	*Ancestral*regions	*Intermediate*regions	*Newly colonized*regions	*Ancestral*regions	*Intermediate*regions	*Newly colonized*regions
*n* sites	122	83	87	122	83	87	122	83	87
*n* larvae	20,442	10,272	11,974	20,442	10,272	11,974	20,442	10,272	11,974
*SP*	62.30**^a^**	68.67**^a^**	37.93**^b^**	54.92**^a^**	59.04**^a^**	31.03**^b^**	79.51**^a^**	79.52**^a^**	51.72**^a^**
Overall *SR*	5	3	3	6	5	3	11	8	6
Mean *SR* per site	1.57**^a^**	1.21**^a^**	0.52**^b^**	1.61**^a^**	1.00**^b^**	0.52**^b^**	3.18**^a^**	2.21**^a^**	1.04**^b^**
	(1.27)	(0.83)	(0.85)	(1.08)	(0.82)	(0.63)	(2.01)	(1.45)	(1.35)
Mean *PR* per site	3.01**^b^**	5.68**^c^**	1.34**^a^**	0.99**^a,b^**	2.51**^b^**	0.58**^a^**	4.00**^b^**	8.19**^c^**	1.92**^a^**
	(3.44)	(7.83)	(2.42)	(1.65)	(8.45)	(1.20)	(3.79)	(12.33)	(3.15)
Mean *H′* per region	0.68**^a^**	0.63**^a^**	0.49**^a^**	0.47**^a^**	0.40**^a^**	0.53**^a^**	0.92**^a^**	0.71**^a^**	0.67**^a^**
	(0.16)	(0.18)	(0.13)	(0.25)	(0.18)	(0.03)	(0.28)	(0.25)	(0.30)

The indices calculated were % of sites at which at least one ECB larva was infested with a parasitoid (*SP*), parasitism rates (*PR*, mean % ± s.d.), overall species richness (*SR*), mean SR per site (mean ± s.d.) and Shannon and Weaver's diversity index (*H′*, mean bits ± s.d.). Standard deviations (s.d.) are given in brackets. Different letters indicate significant differences between indices in a particular row.

The difference in mean *PR* between *ancestral* and *intermediate* regions was not due to a higher proportion of *SP*. In these two groups of regions, at least one parasitoid was detected at about 80% of the sites. In the *newly colonized* regions, this proportion fell to only about 50%, indicating that about half the sites were parasitoid-free. These differences were true and of similar magnitude for both the tachinids and the hymenopterans (Table 3).

A different situation was observed for *SR*. We found that 11 species were recorded in *ancestral* regions, *versus* eight in *intermediate* regions and six in *newly colonized* regions. This decrease in overall *SR* was entirely due to a loss of species from the *ancestral* regions to the two other groups of regions. Indeed, all species recorded in the *intermediate* and *newly colonized* regions were also recorded in the *ancestral* regions. The three species “lost” during the expansion from *ancestral* to *intermediate* regions were the tachinids *Actia pilipennis* and *Voria ruralis* and the hymenopteran *Pristomerus vulnerator*. These three species were rare in the *ancestral* regions (mean *PR*<0.01%). Their absence in the samples collected in the *intermediate* and *newly colonized* regions may therefore be due to differences in sampling intensity. Twice as many larvae were collected from *ancestral* regions as from either of the other two groups of regions (Table 3). The *newly colonized* regions were further characterised by the loss of another two hymenopteran species: *B. brevicornis* and *M. messoria*. Although these two species were rare in the other two groups of regions (mean *PR* = 0.32%), their absence from all the samples collected from the *newly colonized* regions probably reflects the true absence of these two species of parasitoids.

For all parasitoids considered together, the mean *SR* per site in the *newly colonized* regions was one half to one third that in the *intermediate* and *ancestral* regions, respectively (Tukey's HSD test, *p* = 0.012 and *p*<0.001). Similar findings were obtained for the two groups of parasitoids considered separately (Table 3).

Finally, the mean *H′* per region followed a trend similar to that for mean *SR* per site. For all parasitoids, the highest mean *H′* value was indeed recorded in the *ancestral* regions and the lowest mean value was that for the *newly colonized* regions (Table 3). These differences were only marginally significant (Tukey's HSD test, *p* = 0.058), probably due to a lack of power (see *Materials and Methods* section).

## Discussion

Global warming, pesticide and fertiliser applications, the conversion of natural ecosystem to agricultural fields, modifications to ecosystems or landscapes by urbanization and any other change triggered by human activity induced significant changes within parasitoid communities, particularly among parasitoids infesting agricultural pests [34], [35], [8]. We document here the changes that have occurred in parasitoid communities for the ECB, the main pest of maize, based on two substantial historical datasets. Each of these datasets covered a period of several years and a substantial geographical area, and both involved the sampling of several thousands/millions of host larvae. From 2001 to 2005, parasitism rate (*PR*), species richness (*SR*), percentage of sites infested (*SP*) and Shannon and Weaver diversity index (*H′*) varied between regions, but remained stable from year to year in any given region. A stability of the *PR* across years was also reported by Thompson and Parker [53] and Parker et al. [54] for the sampling campaigns in 1921 to 1928. Thus, differences in abundance, composition and richness between these two periods of time probably result from real and durable changes within the parasitoid communities rather than biased and/or limited sampling.

### Changes in the *Ostrinia* parasitoid community over time

This study provides one of the first descriptions of changes in the parasitoid community over several decades. Parasitoid communities have generally been compared between habitats differing in their levels of urbanization (e.g., [27], [35]), landscape structuring (e.g., [25]) and soil fertility (e.g., [36]). These previous studies have investigated the influence of a particular factor on species diversity. However, they were unable to explore the stability of such changes over time. Such an exploration was carried out here for the ECB. For this species, the last 80 years of the 20^th^ century were marked by a decrease in the *PR* due to parasitoids to about two thirds its initial value. *SR* was unaffected, because the parasitoid community was marked by a well balanced loss and gain of species. The picture was strikingly different for *O. scapulalis*, a sibling species of the ECB. The mean *PR* of this species for all types of parasitoid did not decline between the two study periods. Conversely, the parasitoid community of this species probably suffered a decrease in *SR*, with the loss of at least one species during the course of the 20^th^ century and expansion of the range of one braconid wasp, *M. cingulum*, which accounted for >99% of the parasitoids emerging from diapausing ECB larvae.

The ECB evolved in a cultivated agro-ecosystem that has seen considerable changes over the last 80 years. This period has indeed been marked by the introduction of insecticides and herbicides, new maize varieties and combine harvesters. However, these factors have had no significant effect on the densities of *O. nubilalis* larvae feeding on this crop. The number of larvae per stalk reported by Thompson and Parker [53] for the 1921–1925 period was similar to that we recorded during the last 10 years overall France (DB, MD, LF, NE and AW pers. obs). Similarly, the densities of *O. scapulalis* infesting mugwort stands were similar in the 1920s and the 2000s [57]. Furthermore, mugwort is a common perennial weed that may actually benefit from landscape modifications. Indeed, this species is tolerant to chemical treatments and various management strategies due to its extensive underground network of rhizomes, and is thus an invasive species commonly infesting roadsides, wasteland and agronomic landscapes [60]. The area under maize either remained similar (for Aquitaine) or increased (in the other five regions) during the 20^th^ century [55], [61]. The decrease in *PR* and *SR* within the parasitoid communities of *O. scapulalis* and *O. nubilalis* is therefore unlikely to have resulted from a change in larval density in these hosts or from a decline in the abundance of the host plants of these two pests. This was a distinct possibility, because the *PR* of several species, including parasitoids such as *E. terebrans* ([62] but see [63]) recovered in this study, are host density-dependent [64].

The use of pesticides both on maize and on adjoining fields, often affecting the margins of maize fields, has a limited impact on *O. nubilalis* density but may directly affect the survival of adult parasitoids [65]. More importantly, agricultural landscapes and their surroundings have been profoundly shaped by the use of herbicides, which strongly reduced the abundance and diversity of weeds [66]. In France, the diversity and abundance of weeds have been strongly reduced in maize field borders [67]. Yet these weeds are beneficial to parasitoids by hosting alternative and complementary hosts or by providing food (pollen and nectar) and resting sites to adults [65]. The results obtained by Pavuk and Stinner [62] were inconclusive as to whether weeds within maize field increase ECB parasitism by *E. terebrans*, but data from Landis and Haas [63] suggest that *PR* by this species was significantly influenced by local landscape structure, including proximity of particular noncrop habitats.

The parasitoids infesting the ECB were affected differently over the course of the 20^th^ century, with the tachinid community displaying greater stability than the hymenopteran community. Combining data from 15 geographically dispersed databases, Stireman et al. [34] found that the *PR* of lepidopteran larvae decreased with increasing climatic variability. As in our study, this decrease was more pronounced for hymenopterans than for tachinids. Denys and Schmidt [18] also found that hymenopteran parasitoids were more affected than generalist predators by the isolation resulting from urbanization.

The higher stability of the tachinid community than of the hymenopteran community is often attributed to the tendency of tachinids to be more generalist [68]. Hymenopteran parasitoids are indeed more host-specific than tachinids [69], [70]. By exploiting various hosts that might individually respond to human changes in different ways, generalists are less likely to be susceptible to landscape modifications [34]. In our case, the lower *PR* for *O. nubilalis* and the loss of species for *O. scapulalis* may not be linked to the host spectrum. For example, one of the two main species of tachinids is almost monophagous (*L. thompsoni* infests only 3 to 4 species [71], [72]), whereas the other is highly polyphagous (*P. nigrolineata* has been recorded on more than 50 hosts from nine different families [73]). The *PR* values of these two species were either unaffected (in Aquitaine) or affected to a similar extent (in Provence-Alpes-Côte-d'Azur and Rhône-Alpes). Similarly, *M. cingulum* parasitises almost exclusively the genus *Ostrinia* [74], [75], whereas *A. thompsoni* and *M. messoria* are oligophagous and polyphagous, respectively [53]. Decreases in *PR* and species richness may instead reflect the need for complementary hosts. For instance, the maintenance and expansion of *M. cingulum* on *O. scapulalis* may be due to its ability to complete its life cycle entirely on this host. Conversely, adults of the other two principal species of hymenopterans recorded during the 1920s and from the samples collected in the 2000s, *A. thompsoni* and *M. messoria*, emerge during the winter, well before the occurrence of *O. scapulalis* larvae, and therefore probably require complementary hosts for the maintenance of intermediate generation [53]. As indicated above, the decrease in abundance and diversity of weeds due to the use of herbicides (e.g., [66]) may have reduced the availability of those complementary hosts [65].

This explanation probably applies to only some species. For instance, the decrease in the rate of parasitism by *S. turionum* in ECB populations may be linked to climate change and notably to the global warming. Based on the data provided by Météo France (http://france.meteofrance.com/) we have calculated that the temperature in France – compared to the period 1971–2000 – was (mean ± standard error) −0.63±0.03°C and 0.64±0.11°C during the periods 1921–1928 and 2001–2005 respectively. Thompson and Parker [53] never found *S. turionum* in Provence-Alpes-Côte-d'Azur and, based on climographs, they suggested that *S. turionum* could not thrive in districts with hot, dry summers, such as those of the Mediterranean coastal area. During the 2001–2005 period, *S. turionum* was not only absent from Provence-Alpes-Côte-d'Azur and Languedoc-Roussillon, another Mediterranean coastal region, but it was also almost entirely absent from the other southern and eastern regions, in which it was highly abundant in the 1920s. During the 2000s, rates of parasitism by *S. turionum* were higher in the cooler central, western and northern regions than in the warmer southern and eastern regions. Thus, both changes in *PR* since the 1920s and the current *PR* and spatial distribution across France suggest a direct influence of global warming on *S. turionum*.

### Changes in the ECB parasitoid community during expansion of its range

Regions colonized by ECB during the 1950s had a *PR* and an *SR*, lower than those of the *ancestral* regions by factors of two and three, respectively. This pattern was observed for both tachinids and hymenopterans. The regions located between the *ancestral* and *newly colonized* regions had a slightly and not significantly lower *SR* than these regions but, surprisingly, had a higher *PR* than the *ancestral* regions.

The observed decrease in *PR* is consistent with findings for many invasive species, which are often parasitised to a lesser extent in their area of introduction than in their native areas [41], [40], [76], [77]. This pattern is also consistent with that for two other species with a range that expanded in the UK during the 20^th^ century: the gall wasp *Andricus kollari* [48] and the leaf mining moth *Phyllonorycter leucographella* [50]. For these two species, parasitoid *SR* and *PR* declined with latitude towards the current edge of their range. These decreases in *PR* in recently invaded areas may have several causes, including a lag time between host expansion and the establishment of parasitoids, a lower fitness of the parastoid than of the host in the introduced areas and the absence of other required/alternative hosts in new locations.

In western and northern France, *O. nubilalis* became fully sympatric with *O. scapulalis* feeding on mugwort after its expansion. The current ranges of mugwort, which is a very common weed, and maize, which is now widely cultivated throughout France [61], together with the density of larvae on these two plants, provided many opportunities for parasite exchange between the parasitoid communities infesting these two sibling *Ostrinia* species. In addition to this interaction between the *O. scapulalis* and *O. nubilalis* communities, *O. nubilalis*, which is present in very large numbers over very large areas, may have acquired native parasitoid species from other hosts acting as reservoirs/refuges, during its expansion.

Kelly et al. [44] reviewed published findings for animal parasites and showed that native species account for 67% of the parasite fauna of non-indigenous animals from a range of taxonomic groups. Such phenomena have been reported principally for mammals and birds, with only a few examples for phytophagous insects. One reason for this is that few parasitoids are thought to attack host insects from different feeding guilds, because different parasitism strategies are required for the use of hosts with different feeding patterns, including the use of different host plants. Sugiura [78] tested this hypothesis and found that few dipteran and hymenopteran parasitoids were common to phytophagous insects from different feeding guilds. However, many leaf mining moths in newly colonized areas were rapidly adopted as hosts by native parasitoids [79]–[83], [50]. Parasitoid species attacking *Phyllonorycter* spp. have a low host specificity [84], [85], and many of the parasitoids recorded from *Phyllonorycter* are generalists on leafminers, including dipterans and other lepidopterans [86]. This probably facilitates switches from *Phyllonorycter* spp., even when the new host insect feeds on a different host plant. Finally, during its spread northwards to central Scotland, *P. leucographella* switched to a new host plant. Interestingly, this switch was not associated with a change in parasitoid assemblage and rates of parasitism were significantly higher than those for the ancestral host [50].

Our data show that *O. nubilalis* did not capture any native parasitoids during the expansion of its range driven by human activity. Indeed, samples collected from *newly colonized* regions included no species absent from the *ancestral* regions. Two hymenopteran species, *B. brevicornis* and *M. messoria*, seem to have been “lost” during the colonisation process. Our data also show that the expansion of the range of ECB induced no particular parasite spill-over or spill-back toward *O. scapulalis*. Indeed, the principal, and in some cases only, parasitoid of *O. scapulalis*, *M. cingulum*, did not shift to *O. nubilalis*. Similarly, *A. thompsoni* and *M. messoria*, which were frequently found on *O. scapulalis* in northern regions during the 1920s, were not recorded on *O. nubilalis* at any great frequency during the 2001–2005 period. Finally, *O. nubilalis* brought no new parasitoids to the community infesting *O. scapulalis* on mugwort.

The introduction of maize into Europe five centuries ago provided *O. nubilalis* with an enemy-free environment in which to thrive [59]. Taking into account all the regions and parasite species studied here, *O. nubilalis* has experienced lower *PR* than its sibling and putative ancestral species, *O. scapulalis*. We also show here that the parasitoid load of *O. nubilalis* has further decreased over time, probably due to changes driven by human activity, and over space, with the human-driven westward and northward expansion of the range of this species. Indeed, these expansions were associated with a release of the parasitoid species associated with *O. nubilalis*. Meanwhile, *O. nubilalis* did not pick up any of the native parasitoids evolving in other host species in western and northern France. These two phenomena enhanced the difference in *PR* between *O. nubilalis* and *O. scapulalis* in the regions in which they are sympatric, potentially contributing to their genetic divergence.

## Materials and Methods

### Dataset for 2001 to 2005

From 2001 to 2005, the extension services of the French Ministry of Agriculture and the *Fédérations Régionales de Défense contre les Organismes Nuisibles* surveyed maize (*Zea mays* L.) fields at 292 sites in 19 of the 22 regions of France: 75 in 2001, 48 in 2002, 54 in 2003, 53 in 2004, and 62 in 2005 (Table S2). Sites (each >1 ha) were chosen such that the maize fields sampled (i) were free of any treatments (either insecticides or biological control) against ECB, (ii) were representative of the climatic variability for each region, (iii) contained similar numbers of early- and late-sown maize fields, (iv) were heavily infested with ECB larvae.

We collected seven or eight samples per site, giving a total of 2,154 samples, yielding 42,688 ECB larvae, for all sites and all years. At each site, samples were taken at regular intervals from July, when the first L3 ECB larvae were observed in maize stalks, until October, when the maize was harvested. During each sampling session, we collected ECB larvae from five to 20 plots, each plot consisting of 10 consecutive maize plants (stalks and ears). The ECB larvae from each sample were placed in a single 55×40×6 mm ventilated box for the rearing and emergence of parasitoids. In each of the 2,154 boxes, we collected all the parasitoids that successfully emerged, from the date of sampling until the following summer. Boxes were checked and cleaned (with 70% alcohol) every two weeks, or every 7–10 days during periods in which the climatic conditions were favourable for the development of particular ECB stages. ECB larvae were fed maize stalk and ears. During the winter, corrugated cardboard was introduced into the boxes to enable the larvae to overwinter. Each parasitoid was recorded and identified to species level on the basis of its morphological characters.

For the 2000s period we also used some data published by Pélissié et al. [59]. These authors provided *PR* values for *O. scapulalis* collected on mugwort at seven locations in four regions of France. Althoug their data was limited to diapausing larvae, a particular stage of larval development, in a single year (2002) and concerned only a small number of larvae (*n* = 727), the *PR* values could be compared to those recorded on the same host plant during the USDA campaigns in 1921 to 1928.

### Dataset for 1921 to 1928

ECB parasitism by parasitoids in France was explored by USDA teams during extraordinary yearly campaigns of sampling in Europe and the Far East, between 1920 and 1937 [52]. Over this time period, more than 23 million *Ostrinia* larvae were forwarded from Europe to the United States. Unfortunately, Baker et al. [52] provided no details concerning the regions and host plants of origin of these larvae, but Thompson and Parker [53] and Parker et al. [54] provided parasitism rates for eight regions of France including a precise record of the host plants from which the ECB larvae were collected, for the periods 1921–1925 and 1926–1928.

### Regions of ancestral, intermediate and new colonisation by ECB

**Figure 4:**
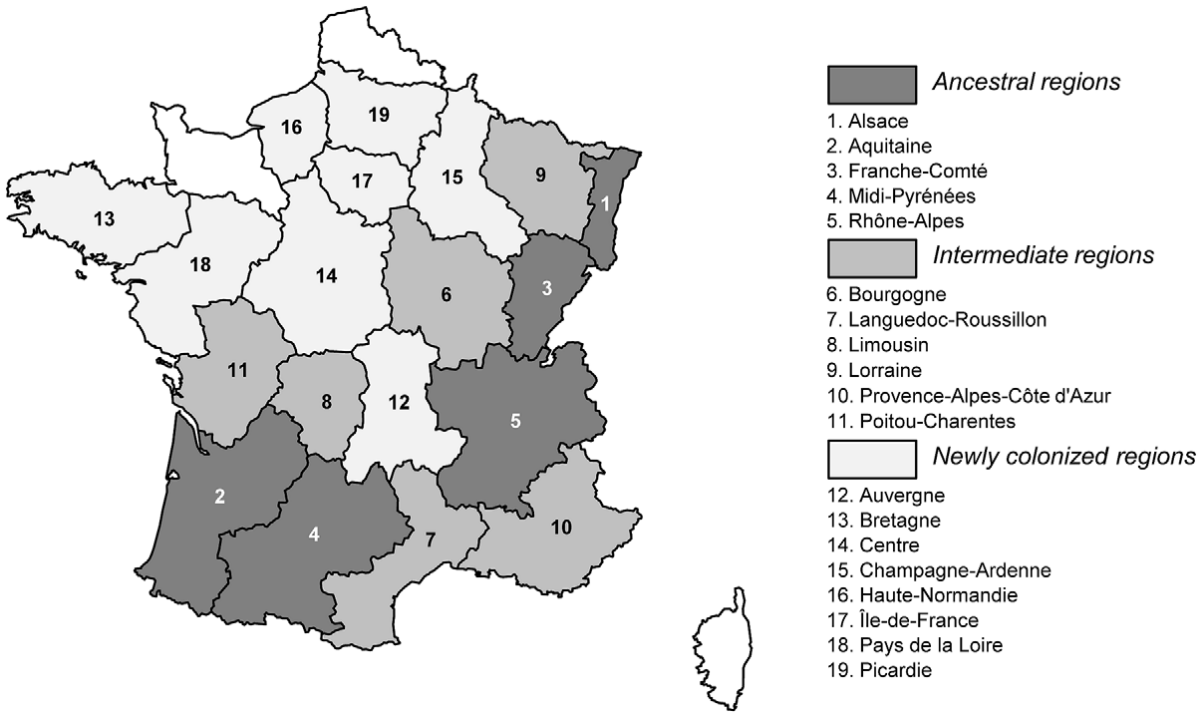
Geographical location of the *ancestral*, *intermediate* and *newly colonized* regions infested with ECB in France.

The 22 regions of France can be subdivided into three groups according to their history of maize cultivation and, thus, of ECB infestation (Figure 4): (i) *ancestral regions* in which maize was already widely cultivated in 1920s. These regions are located in two distinct geographical areas: one in south-western France, including Aquitaine and Midi-Pyrénées and the other in eastern France, including Alsace, Franche-Comté and Rhône-Alpes. According to Thompson and Parker [53], Faucher [55] and Grenier et al. [87] maize cultivation was long restricted to these two areas, until the selection of new varieties of maize permitted its expansion in the 1950s (ii) *intermediate regions* geographically connected to *ancestral regions*. These include Bourgogne, Languedoc-Roussillon, Limousin, Lorraine, Provence-Alpes-Côte-d'Azur and Poitou-Charentes. In these regions, maize was cultivated but covered only a very small areas (<1% of the total agricultural area) before the 1920s (see Figure 1 in [55]). The area under this crop probably increased before the World War II and these areas were certainly colonized by ECB between 1930 and 1950 and (iii) *newly colonized regions*, including Auvergne, Bretagne, Centre, Champagne-Ardenne, Haute-Normandie, Ile-de-France, Pays de la Loire and Picardie. In these regions, maize was grown very rarely, if at all, before the 1950s [55], [87]. According to Thompson and Parker [53], the rare maize fields cultivated in Ile-de-France during the 1920s were free of ECB infestations. Hence, even if rare maize fields were cultivated in these areas during the 1920s, ECB probably did not colonise these regions before the massive expansion of maize cultivation in the 1950s.

### Data analysis

#### Dataset for 2001–2005

For each site, for each species and for all parasitoids considered together, we calculated (i) the parasitism rate (*PR*), using the following formula:
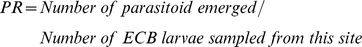
and (ii) species richness (*SR*) corresponding to the number of different species among the parasitoids emerging from the ECB larvae collected from this site.

For each region, and for each of the five years, we calculated (i) a mean *PR* per site, (ii) a mean *SR* per region, (iii) the percentage of sites parasitized (*SP*). A site was considered to be parasitized if at least one parasitoid emerged from the seven to eight samples collected at this site, and (iv) the Shannon and Weaver [88] index *H′*, using the following formula:
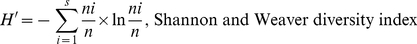
(1)where *n* and *ni* are the total number of parasitoids and the number of parasitoids species *i*, respectively, at all the sites located in this region.

#### Dataset for 1921–1928

Thompson and Parker [53] and Parker et al. [54] gave a mean *PR* per region and per year. As indicated above, they did not specify the number of sites or the number of larvae. We therefore estimated *H′* for each region with a modified version of the Shannon and Weaver index:
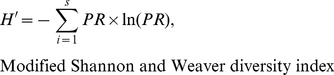
(2)


#### Statistical comparisons

For the 2001 to 2005 dataset, the effects of region (or groups of regions – i.e., *ancestral*, *intermediate* and *newly colonized* regions) and year, together with the interaction between these two factors, on mean *PR*, mean *SR*, *SP* and *H′* were assessed with a generalised linear model (GLM, [89]). *PR* values were subjected to an arcsine square root transformation to normalise their distribution. We used Gaussian (for *PR* and *H′*), binomial negative (for *SR*) or binomial (for *SP*) models (error distributions) associated with the Akaike information criterion [90], in the open-source software package R ([91], version 2.11.1). Within a given region, we used *t*-tests to compare the mean *PR*, mean *SR* and mean *H′* between the two periods of time: 1921–1928 and 2001–2005. Finally, we used Tukey's HSD test for multiple comparisons of means of *PR*, *SR*, *SP*, *H′* between groups of regions in 2001–2005.

## Supporting Information

Table S1 Taxonomic synonymies between the studies performed during the 1920's and the 2000's.
(DOC)

Table S2 Parasitism rates (%) overall parasitoids and for each tachinid species infesting *O. nubilalis* collected on maize from 2001 to 2005.
(DOC)

Table S3 Parasitism rates (%) of the hymenopteran species infesting *O. nubilalis* collected on maize from 2001 to 2005.
(DOC)

Table S4 Results of GLM analyses testing the “region”, “year” and the interaction “region x year” effects on mean parasitism rate (*PR*) per site and on % of infested sites. Values of Fisher (*F*) and Chi-square (*χ^2^*) are given for the gaussian and binomial models, respectively. *df* = degree of fredoom.
(DOC)

Table S5 Results of GLM analyses testing the “region groups”, “year” and the interaction “region groups x year” effects on mean species richness (*SR*) per region, mean parasitism rate (*PR*) per site, Shannon and Weaver diversity Index (*H′*) per region and % infested sites. Values of Fisher (*F*) and Chi-square (*χ^2^*) are given for the gaussian and binomial/binomial negative models, respectively. *df* = degree of fredoom.
(DOC)

Table S6 Parasitism rates (in %) overall tachinids, overall hymenopteran and overall parasitoids infesting *O. nubilalis* and *O. scapulalis*. References: A = Thompson & Parker (1928), B = Paillot (1928), C = Parker et al. (1929), D = Pélissié et al. (2010), E = this study. Sd = standard deviation. * Not given but probably several thousands, ** Not given but probably several.
(DOC)

Table S7 Parasitism rates (in %) of the three main tachinid species infesting *O. nubilalis* and *O. scapulalis*. Parasitism rates of *Actia pilipennis*, *Eumea mitis*, *Nemorilla maculosa*, *Voria ruralis* and *Zenillia mitis* were negligible and are therefore not given. References: A = Thompson & Parker (1928), B = Paillot (1928), C = Parker et al. (1929), D = Pélissié et al. (2010), E = this study. Sd = standard deviation. * Not given but probably several thousands, ** Not given but probably several.
(DOC)

Table S8 Parasitism rates (in %) of the hymenopteran Braconidae infesting *O. nubilalis* and *O. scapulalis*. Parasitism rates of Eulophidae were negligible, and thus not given. References: A = Thompson & Parker (1928), B = Paillot (1928), C = Parker et al. (1929), D = Pélissié et al. (2010), E = this study. Sd = standard deviation. * Not given but probably several thousands, ** Not given but probably several.
(DOC)

Table S9 Parasitism rates (in %) of the hymenopteran Ichneumonidae infesting *O. nubilalis* and *O. scapulalis*. Parasitism rates of *Campoplex lugubrinus*, *Campoplex rothi*, *Exeristes roborator*, *Pristomerus vulnerator* and *Theronia atalantae* were negligible and are therefore not given. References: A = Thompson & Parker (1928), B = Paillot (1928), C = Parker et al. (1929), D = Pélissié et al. (2010), E = this study. Sd = standard deviation. * Not given but probably several thousands, ** Not given but probably several.
(DOC)
