# Supplementary material for: Changes in Parasitoid Communities Over Time and Space: A Historical Case Study of the Maize Pest Ostrinia nubilalis
Source: PLoS One. 2011 Sep 30;6(9):e25374. doi: 10.1371/journal.pone.0025374 (PMC3184128; doi:10.1371/journal.pone.0025374)
Supplement: Table S1 — Taxonomic synonymies between the studies performed during the 1920's and the 2000's. (DOC) [file pone.0025374.s001.doc]

**Table S1** – Taxonomic synonymies between the studies performed during the 1920’s and the 2000’s.

| **Family** | **1920’s nomenclature** |  | **2000’s nomenclature** |
| --- | --- | --- | --- |
| Tachinidae | *Masicera senilis* Meigen, 1861 |  | *Lydella thompsoni* Herting, 1959 |
|  | *Zenillia roseanae* Brauer & Bergenstamm, 1891 |  | *Pseudoperichaeta nigrolineata* Walker, 1853 |
| Braconidae | *Microgaster tibialis* Nees, 1834 |  | *Microgaster messoria*  Haliday, 1834 |
| Ichneumonidae | *Dioctes punctoria* Roman, 1923 |  | *Eriborus terebrans* Gravenhorst, 1829 |
|  | *Eulimneria crassifemur* Thomson, 1887 |  | *Sinophorus turionum* Ratzeburg, 1844 |
