# Supplementary material for: Changes in Parasitoid Communities Over Time and Space: A Historical Case Study of the Maize Pest Ostrinia nubilalis
Source: PLoS One. 2011 Sep 30;6(9):e25374. doi: 10.1371/journal.pone.0025374 (PMC3184128; doi:10.1371/journal.pone.0025374)
Supplement: Table S2 — Parasitism rates (%) overall parasitoids and for each tachinid species infesting O. nubilalis collected on maize from 2001 to 2005. (DOC) [file pone.0025374.s002.doc]

**Table S2** – Parasitism rates (%) overall parasitoids and for each tachinid species infesting *O. nubilalis* collected on maize from 2001 to 2005.

|  |  |  |  | **Overall parasitism** | |  | ***Actia pilipennis*** | |  | ***Lydella thompsoni*** | |  | ***Pseudoperichaeta nigrolineata*** | |  | ***Pseudoperichaeta palesoidea*** | |  | ***Voria***  ***ruralis*** | |
| --- | --- | --- | --- | --- | --- | --- | --- | --- | --- | --- | --- | --- | --- | --- | --- | --- | --- | --- | --- | --- |
| **Region** | **Year** | **N sites** | **N larvae** | **mean** | **max** |  | **mean** | **max** |  | **mean** | **max** |  | **mean** | **max** |  | **mean** | **max** |  | **mean** | **max** |
| Alsace | 2001 | 5 | 731 | 0.00 | - |  | 0.00 | - |  | 0.00 | - |  | 0.00 | - |  | 0.00 | - |  | 0.00 | - |
|  | 2002 | 5 | 975 | 0.31 | 1.01 |  | 0.00 | - |  | 0.20 | 0.52 |  | 0.00 | - |  | 0.00 | - |  | 0.00 | - |
|  | 2003 | 7 | 1,372 | 2.52 | 8.76 |  | 0.00 | - |  | 0.80 | 5.61 |  | 0.00 | - |  | 0.00 | - |  | 0.00 | - |
|  | 2004 | 5 | 999 | 3.00 | 10.53 |  | 0.00 | - |  | 2.90 | 10.53 |  | 0.00 | - |  | 0.00 | - |  | 0.00 | - |
|  | 2005 | 6 | 1,406 | 0.26 | 0.44 |  | 0.00 | - |  | 0.00 | - |  | 0.00 | - |  | 0.00 | - |  | 0.00 | - |
| Aquitaine | 2001 | 5 | 783 | 8.23 | 16.24 |  | 0.00 | - |  | 3.46 | 9.49 |  | 3.69 | 10.66 |  | 0.00 | - |  | 0.00 | - |
|  | 2002 | 5 | 614 | 5.61 | 14.55 |  | 0.12 | 0.61 |  | 0.41 | 1.43 |  | 1.52 | 5.45 |  | 0.94 | 2.78 |  | 0.14 | 0.71 |
|  | 2003 | 5 | 879 | 6.31 | 9.70 |  | 0.00 | - |  | 1.16 | 1.82 |  | 4.49 | 7.88 |  | 0.00 | - |  | 0.00 | - |
|  | 2004 | 5 | 776 | 11.01 | 13.79 |  | 0.00 | - |  | 4.14 | 8.16 |  | 4.49 | 6.42 |  | 0.00 | - |  | 0.00 | - |
|  | 2005 | 5 | 834 | 7.58 | 11.18 |  | 0.00 | - |  | 3.30 | 7.89 |  | 2.68 | 6.25 |  | 0.00 | - |  | 0.00 | - |
| Auvergne | 2001 | 2 | 322 | 0.00 | - |  | 0.00 | - |  | 0.00 | - |  | 0.00 | - |  | 0.00 | - |  | 0.00 | - |
|  | 2003 | 2 | 465 | 0.61 | 1.23 |  | 0.00 | - |  | 0.00 | - |  | 0.00 | - |  | 0.00 | - |  | 0.00 | - |
|  | 2004 | 3 | 274 | 0.00 | - |  | 0.00 | - |  | 0.00 | - |  | 0.00 | - |  | 0.00 | - |  | 0.00 | - |
|  | 2005 | 3 | 891 | 0.00 | - |  | 0.00 | - |  | 0.00 | - |  | 0.00 | - |  | 0.00 | - |  | 0.00 | - |
| Bourgogne | 2001 | 3 | 422 | 4.04 | 9.29 |  | 0.00 | - |  | 0.71 | 1.41 |  | 0.00 | - |  | 0.00 | - |  | 0.00 | - |
|  | 2002 | 3 | 345 | 0.29 | 0.83 |  | 0.00 | - |  | 0.00 | - |  | 0.00 | - |  | 0.00 | - |  | 0.00 | - |
|  | 2003 | 2 | 268 | 4.88 | 5.47 |  | 0.00 | - |  | 0.71 | 1.43 |  | 0.00 | - |  | 0.00 | - |  | 0.00 | - |
|  | 2004 | 3 | 388 | 1.48 | 3.01 |  | 0.00 | - |  | 0.24 | 0.71 |  | 0.00 | - |  | 0.00 | - |  | 0.00 | - |
|  | 2005 | 3 | 507 | 0.85 | 1.36 |  | 0.00 | - |  | 0.15 | 0.44 |  | 0.00 | - |  | 0.00 | - |  | 0.00 | - |
| Bretagne | 2001 | 3 | 315 | 0.00 | - |  | 0.00 | - |  | 0.00 | - |  | 0.00 | - |  | 0.00 | - |  | 0.00 | - |
|  | 2002 | 3 | 280 | 0.00 | - |  | 0.00 | - |  | 0.00 | - |  | 0.00 | - |  | 0.00 | - |  | 0.00 | - |
|  | 2003 | 0 | 317 | 1.90 | 2.97 |  | 0.00 | - |  | 0.00 | - |  | 0.00 | - |  | 0.00 | - |  | 0.00 | - |
|  | 2004 | 3 | 380 | 0.00 | - |  | 0.00 | - |  | 0.00 | - |  | 0.00 | - |  | 0.00 | - |  | 0.00 | - |
|  | 2005 | 3 | 353 | 0.00 | - |  | 0.00 | - |  | 0.00 | - |  | 0.00 | - |  | 0.00 | - |  | 0.00 | - |
| Centre | 2001 | 6 | 336 | 6.95 | 13.16 |  | 0.00 | - |  | 3.15 | 7.89 |  | 1.55 | 8.33 |  | 0.00 | - |  | 0.00 | - |
|  | 2003 | 1 | 102 | 0.00 | - |  | 0.00 | - |  | 0.00 | - |  | 0.00 | - |  | 0.00 | - |  | 0.00 | - |
|  | 2004 | 2 | 333 | 0.00 | - |  | 0.00 | - |  | 0.00 | - |  | 0.00 | - |  | 0.00 | - |  | 0.00 | - |
|  | 2005 | 5 | 705 | 0.00 | - |  | 0.00 | - |  | 0.00 | - |  | 0.00 | - |  | 0.00 | - |  | 0.00 | - |
| Champagne-Ardenne | 2001 | 3 | 293 | 1.46 | 2.50 |  | 0.00 | - |  | 0.00 | - |  | 0.00 | - |  | 0.00 | - |  | 0.00 | - |
|  | 2002 | 2 | 227 | 3.03 | 6.06 |  | 0.00 | - |  | 0.38 | 0.76 |  | 0.00 | - |  | 0.00 | - |  | 0.00 | - |
|  | 2003 | 2 | 237 | 1.01 | 2.02 |  | 0.00 | - |  | 0.00 | - |  | 0.00 | - |  | 0.00 | - |  | 0.00 | - |
|  | 2004 | 2 | 220 | 0.54 | 1.09 |  | 0.00 | - |  | 0.00 | - |  | 0.00 | - |  | 0.00 | - |  | 0.00 | - |
|  | 2005 | 2 | 294 | 0.00 | - |  | 0.00 | - |  | 0.00 | - |  | 0.00 | - |  | 0.00 | - |  | 0.00 | - |
| Franche-Comté | 2001 | 3 | 256 | 0.00 | - |  | 0.00 | - |  | 0.00 | - |  | 0.00 | - |  | 0.00 | - |  | 0.00 | - |
|  | 2004 | 3 | 383 | 1.04 | 2.17 |  | 0.00 | - |  | 0.00 | - |  | 0.00 | - |  | 0.00 | - |  | 0.00 | - |
|  | 2005 | 3 | 330 | 0.28 | 0.83 |  | 0.00 | - |  | 0.00 | - |  | 0.00 | - |  | 0.00 | - |  | 0.00 | - |
| Haute-Normandie | 2001 | 1 | 189 | 0.00 | - |  | 0.00 | - |  | 0.00 | - |  | 0.00 | - |  | 0.00 | - |  | 0.00 | - |
|  | 2004 | 1 | 221 | 3.17 | - |  | 0.00 | - |  | 1.81 | - |  | 0.00 | - |  | 0.00 | - |  | 0.00 | - |
|  | 2005 | 1 | 278 | 2.88 | - |  | 0.00 | - |  | 2.52 | - |  | 0.00 | - |  | 0.00 | - |  | 0.00 | - |
| Ile-de-France | 2002 | 1 | 69 | 1.45 | - |  | 0.00 | - |  | 0.00 | - |  | 0.00 | - |  | 0.00 | - |  | 0.00 | - |
|  | 2003 | 2 | 124 | 0.00 | - |  | 0.00 | - |  | 0.00 | - |  | 0.00 | - |  | 0.00 | - |  | 0.00 | - |
|  | 2004 | 1 | 105 | 0.00 | - |  | 0.00 | - |  | 0.00 | - |  | 0.00 | - |  | 0.00 | - |  | 0.00 | - |
|  | 2005 | 1 | 161 | 2.48 | - |  | 0.00 | - |  | 2.48 | - |  | 0.00 | - |  | 0.00 | - |  | 0.00 | - |
| Languedoc-Roussillon | 2001 | 2 | 142 | 0.81 | 1.61 |  | 0.00 | - |  | 0.00 | - |  | 0.00 | - |  | 0.00 | - |  | 0.00 | - |
|  | 2002 | 1 | 112 | 2.68 | - |  | 0.00 | - |  | 0.89 | - |  | 1.79 | - |  | 0.00 | - |  | 0.00 | - |
|  | 2003 | 1 | 86 | 3.49 | - |  | 0.00 | - |  | 0.00 | - |  | 1.16 | - |  | 0.00 | - |  | 0.00 | - |
|  | 2004 | 1 | 128 | 3.13 | - |  | 0.00 | - |  | 0.78 | - |  | 0.78 | - |  | 0.00 | - |  | 0.00 | - |
|  | 2005 | 1 | 132 | 0.00 | - |  | 0.00 | - |  | 0.00 | - |  | 0.00 | - |  | 0.00 | - |  | 0.00 | - |
| Limousin | 2001 | 4 | 490 | 17.51 | 28.32 |  | 0.00 | - |  | 10.02 | 24.78 |  | 5.91 | 10.11 |  | 0.00 | - |  | 0.00 | - |
|  | 2002 | 3 | 298 | 25.61 | 47.37 |  | 0.00 | - |  | 24.22 | 45.26 |  | 0.69 | 2.06 |  | 0.00 | - |  | 0.00 | - |
|  | 2003 | 3 | 361 | 0.76 | 1.44 |  | 0.00 | - |  | 0.00 | - |  | 0.00 | - |  | 0.00 | - |  | 0.00 | - |
|  | 2004 | 3 | 321 | 16.45 | 24.51 |  | 0.00 | - |  | 9.24 | 15.69 |  | 4.05 | 5.66 |  | 0.00 | - |  | 0.00 | - |
|  | 2005 | 3 | 327 | 25.23 | 40.51 |  | 0.00 | - |  | 14.23 | 26.58 |  | 5.53 | 10.45 |  | 0.00 | - |  | 0.00 | - |
| Lorraine | 2002 | 4 | 276 | 0.00 | - |  | 0.00 | - |  | 0.00 | - |  | 0.00 | - |  | 0.00 | - |  | 0.00 | - |
|  | 2003 | 4 | 186 | 31.96 | 70.97 |  | 0.00 | - |  | 4.84 | 19.35 |  | 0.00 | - |  | 0.00 | - |  | 0.00 | - |
|  | 2005 | 4 | 452 | 0.22 | 0.89 |  | 0.00 | - |  | 0.22 | 0.89 |  | 0.00 | - |  | 0.00 | - |  | 0.00 | - |
| Midi-Pyrénées | 2001 | 10 | 1,502 | 6.83 | 13.48 |  | 0.00 | - |  | 1.41 | 3.08 |  | 4.73 | 10.64 |  | 0.00 | - |  | 0.00 | - |
|  | 2002 | 5 | 609 | 5.30 | 6.62 |  | 0.00 | - |  | 1.43 | 3.42 |  | 3.20 | 5.15 |  | 0.00 | - |  | 0.00 | - |
|  | 2003 | 5 | 1,054 | 4.35 | 8.56 |  | 0.00 | - |  | 2.63 | 6.31 |  | 1.27 | 4.02 |  | 0.00 | - |  | 0.00 | - |
|  | 2004 | 5 | 857 | 5.65 | 12.00 |  | 0.00 | - |  | 1.01 | 3.33 |  | 2.93 | 4.00 |  | 0.24 | 0.67 |  | 0.00 | - |
|  | 2005 | 5 | 789 | 4.76 | 7.30 |  | 0.00 | - |  | 0.87 | 1.81 |  | 2.72 | 4.38 |  | 0.44 | 2.19 |  | 0.00 | - |
| Pays de La Loire | 2001 | 7 | 983 | 3.29 | 9.23 |  | 0.00 | - |  | 2.59 | 9.23 |  | 0.30 | 1.46 |  | 0.00 | - |  | 0.00 | - |
|  | 2002 | 5 | 764 | 1.63 | 3.13 |  | 0.00 | - |  | 1.49 | 3.13 |  | 0.00 | - |  | 0.00 | - |  | 0.00 | - |
|  | 2003 | 5 | 707 | 5.35 | 9.20 |  | 0.00 | - |  | 4.14 | 7.08 |  | 0.22 | 1.12 |  | 0.00 | - |  | 0.00 | - |
|  | 2004 | 5 | 882 | 4.53 | 7.50 |  | 0.00 | - |  | 3.08 | 6.10 |  | 0.22 | 1.09 |  | 0.28 | 0.83 |  | 0.00 | - |
|  | 2005 | 5 | 1,059 | 4.12 | 14.36 |  | 0.00 | - |  | 2.22 | 7.73 |  | 0.44 | 1.66 |  | 0.00 | - |  | 0.00 | - |
| Picardie | 2005 | 1 | 88 | 0.00 | - |  | 0.00 | - |  | 0.00 | - |  | 0.00 | - |  | 0.00 | - |  | 0.00 | - |
| Poitou-Charentes | 2001 | 10 | 1,141 | 9.08 | 14.89 |  | 0.00 | - |  | 5.42 | 9.57 |  | 2.71 | 6.00 |  | 0.00 | - |  | 0.00 | - |
|  | 2002 | 5 | 762 | 6.00 | 8.93 |  | 0.00 | - |  | 3.95 | 5.95 |  | 0.74 | 1.71 |  | 0.00 | - |  | 0.00 | - |
|  | 2003 | 5 | 709 | 6.50 | 10.58 |  | 0.00 | - |  | 3.29 | 6.73 |  | 2.09 | 3.45 |  | 0.00 | - |  | 0.00 | - |
|  | 2004 | 5 | 778 | 8.08 | 11.49 |  | 0.00 | - |  | 3.34 | 5.52 |  | 3.12 | 7.47 |  | 0.34 | 1.72 |  | 0.00 | - |
|  | 2005 | 5 | 755 | 5.84 | 11.19 |  | 0.00 | - |  | 3.56 | 8.39 |  | 1.24 | 2.10 |  | 0.00 | - |  | 0.00 | - |
| Provence-Alpes-Côte d'Azur | 2001 | 1 | 103 | 0.00 | - |  | 0.00 | - |  | 0.00 | - |  | 0.00 | - |  | 0.00 | - |  | 0.00 | - |
|  | 2002 | 1 | 103 | 7.77 | - |  | 0.00 | - |  | 1.94 | - |  | 0.00 | - |  | 0.00 | - |  | 0.00 | - |
|  | 2003 | 1 | 362 | 2.49 | - |  | 0.00 | - |  | 1.38 | - |  | 0.00 | - |  | 0.00 | - |  | 0.00 | - |
|  | 2004 | 1 | 171 | 5.85 | - |  | 0.00 | - |  | 4.09 | - |  | 0.00 | - |  | 0.00 | - |  | 0.00 | - |
|  | 2005 | 1 | 147 | 8.84 | - |  | 0.00 | - |  | 6.12 | - |  | 0.00 | - |  | 0.00 | - |  | 0.00 | - |
| Rhône-Alpes | 2001 | 10 | 1,618 | 1.29 | 4.05 |  | 0.00 | - |  | 0.25 | 1.27 |  | 0.00 | - |  | 0.00 | - |  | 0.00 | - |
|  | 2002 | 5 | 787 | 7.37 | 12.10 |  | 0.00 | - |  | 2.54 | 6.06 |  | 0.25 | 0.69 |  | 0.00 | - |  | 0.00 | - |
|  | 2003 | 5 | 555 | 4.36 | 9.01 |  | 0.00 | - |  | 1.74 | 5.41 |  | 0.35 | 0.90 |  | 0.00 | - |  | 0.00 | - |
|  | 2004 | 5 | 1,446 | 6.31 | 10.37 |  | 0.00 | - |  | 3.50 | 5.18 |  | 0.44 | 1.52 |  | 0.06 | 0.30 |  | 0.00 | - |
|  | 2005 | 5 | 887 | 2.82 | 5.94 |  | 0.00 | - |  | 0.00 | - |  | 0.00 | - |  | 0.00 | - |  | 0.00 | - |
