# Supplementary material for: Changes in Parasitoid Communities Over Time and Space: A Historical Case Study of the Maize Pest Ostrinia nubilalis
Source: PLoS One. 2011 Sep 30;6(9):e25374. doi: 10.1371/journal.pone.0025374 (PMC3184128; doi:10.1371/journal.pone.0025374)
Supplement: Table S3 — Parasitism rates (%) of the hymenopteran species infesting O. nubilalis collected on maize from 2001 to 2005. (DOC) [file pone.0025374.s003.doc]

**Table S3** – Parasitism rates (%) of the hymenopteran species infesting *O. nubilalis* collected on maize from 2001 to 2005.

|  |  |  |  | ***Sinophorus turionum*** | |  | ***Bracon brevicornis*** | |  | ***Eriborus terebrans*** | |  | ***Microgaster messoria*** | |  | ***Diadegma fenestrale*** | |  | ***Pristomerus vulnerator*** | |
| --- | --- | --- | --- | --- | --- | --- | --- | --- | --- | --- | --- | --- | --- | --- | --- | --- | --- | --- | --- | --- |
| **Region** | **Year** | **N larvae** | **N sites** | **mean** | **max** |  | **mean** | **max** |  | **mean** | **max** |  | **mean** | **max** |  | **mean** | **max** |  | **mean** | **max** |
| Alsace | 2001 | 731 | 5 | 0.00 | - |  | 0.00 | - |  | 0.00 | - |  | 0.00 | - |  | 0.00 | - |  | 0.00 | - |
|  | 2002 | 975 | 5 | 0.00 | - |  | 0.00 | - |  | 0.00 | - |  | 0.00 | - |  | 0.00 | - |  | 0.10 | 0.51 |
|  | 2003 | 1,372 | 7 | 0.00 | - |  | 1.72 | 8.76 |  | 0.00 | - |  | 0.00 | - |  | 0.00 | - |  | 0.00 | - |
|  | 2004 | 999 | 5 | 0.00 | - |  | 0.00 | - |  | 0.00 | - |  | 0.00 | - |  | 0.00 | - |  | 0.00 | - |
|  | 2005 | 1,406 | 6 | 0.19 | 0.44 |  | 0.00 | - |  | 0.00 | - |  | 0.00 | - |  | 0.07 | 0.43 |  | 0.00 | - |
| Aquitaine | 2001 | 783 | 5 | 0.72 | 1.80 |  | 0.00 | - |  | 0.00 | - |  | 0.12 | 0.59 |  | 0.14 | 0.68 |  | 0.00 | - |
|  | 2002 | 614 | 5 | 0.26 | 0.71 |  | 0.00 | - |  | 0.91 | 2.42 |  | 0.00 | - |  | 0.00 | - |  | 0.12 | 0.61 |
|  | 2003 | 879 | 5 | 0.24 | 0.64 |  | 0.00 | - |  | 0.11 | 0.54 |  | 0.00 | - |  | 0.00 | - |  | 0.00 | - |
|  | 2004 | 776 | 5 | 0.98 | 3.45 |  | 0.00 | - |  | 0.17 | 0.86 |  | 0.00 | - |  | 0.00 | - |  | 0.00 | - |
|  | 2005 | 834 | 5 | 0.10 | 0.49 |  | 0.00 | - |  | 0.62 | 1.38 |  | 0.00 | - |  | 0.11 | 0.53 |  | 0.00 | - |
| Auvergne | 2001 | 322 | 2 | 0.00 | - |  | 0.00 | - |  | 0.00 | - |  | 0.00 | - |  | 0.00 | - |  | 0.00 | - |
|  | 2003 | 465 | 2 | 0.00 | - |  | 0.00 | - |  | 0.61 | 1.23 |  | 0.00 | - |  | 0.00 | - |  | 0.00 | - |
|  | 2004 | 274 | 3 | 0.00 | - |  | 0.00 | - |  | 0.00 | - |  | 0.00 | - |  | 0.00 | - |  | 0.00 | - |
|  | 2005 | 891 | 3 | 0.00 | - |  | 0.00 | - |  | 0.00 | - |  | 0.00 | - |  | 0.00 | - |  | 0.00 | - |
| Bourgogne | 2001 | 422 | 3 | 3.33 | 9.29 |  | 0.00 | - |  | 0.00 | - |  | 0.00 | - |  | 0.00 | - |  | 0.00 | - |
|  | 2002 | 345 | 3 | 0.29 | 0.83 |  | 0.00 | - |  | 0.00 | - |  | 0.00 | - |  | 0.00 | - |  | 0.00 | - |
|  | 2003 | 268 | 2 | 3.42 | 4.69 |  | 0.00 | - |  | 0.00 | - |  | 0.00 | - |  | 0.00 | - |  | 0.00 | - |
|  | 2004 | 388 | 3 | 0.25 | 0.75 |  | 0.00 | - |  | 0.00 | - |  | 0.00 | - |  | 0.00 | - |  | 0.00 | - |
|  | 2005 | 507 | 3 | 1.14 | 1.36 |  | 0.00 | - |  | 0.00 | - |  | 0.00 | - |  | 0.00 | - |  | 0.00 | - |
| Bretagne | 2001 | 315 | 3 | 0.00 | - |  | 0.00 | - |  | 0.00 | - |  | 0.00 | - |  | 0.00 | - |  | 0.00 | - |
|  | 2002 | 280 | 3 | 0.00 | - |  | 0.00 | - |  | 0.00 | - |  | 0.00 | - |  | 0.00 | - |  | 0.00 | - |
|  | 2003 | 317 | 0 | 1.28 | 2.97 |  | 0.00 | - |  | 0.00 | - |  | 0.00 | - |  | 0.00 | - |  | 0.00 | - |
|  | 2004 | 380 | 3 | 0.00 | - |  | 0.00 | - |  | 0.00 | - |  | 0.00 | - |  | 0.00 | - |  | 0.00 | - |
|  | 2005 | 353 | 3 | 0.00 | - |  | 0.00 | - |  | 0.00 | - |  | 0.00 | - |  | 0.00 | - |  | 0.00 | - |
| Centre | 2001 | 336 | 6 | 1.13 | 4.17 |  | 0.00 | - |  | 0.00 | - |  | 0.00 | - |  | 0.89 | 2.70 |  | 0.00 | - |
|  | 2003 | 102 | 1 | 0.00 | - |  | 0.00 | - |  | 0.00 | - |  | 0.00 | - |  | 0.00 | - |  | 0.00 | - |
|  | 2004 | 333 | 2 | 0.00 | - |  | 0.00 | - |  | 0.00 | - |  | 0.00 | - |  | 0.00 | - |  | 0.00 | - |
|  | 2005 | 705 | 5 | 0.00 | - |  | 0.00 | - |  | 0.00 | - |  | 0.00 | - |  | 0.00 | - |  | 0.00 | - |
| Champagne-Ardenne | 2001 | 293 | 3 | 1.46 | 2.50 |  | 0.00 | - |  | 0.00 | - |  | 0.00 | - |  | 0.00 | - |  | 0.00 | - |
|  | 2002 | 227 | 2 | 2.65 | 5.30 |  | 0.00 | - |  | 0.00 | - |  | 0.00 | - |  | 0.00 | - |  | 0.00 | - |
|  | 2003 | 237 | 2 | 1.01 | 2.02 |  | 0.00 | - |  | 0.00 | - |  | 0.00 | - |  | 0.00 | - |  | 0.00 | - |
|  | 2004 | 220 | 2 | 0.00 | - |  | 0.00 | - |  | 0.00 | - |  | 0.00 | - |  | 0.00 | - |  | 0.00 | - |
|  | 2005 | 294 | 2 | 0.00 | - |  | 0.00 | - |  | 0.00 | - |  | 0.00 | - |  | 0.00 | - |  | 0.00 | - |
| Franche-Comté | 2001 | 256 | 3 | 0.00 | - |  | 0.00 | - |  | 0.00 | - |  | 0.00 | - |  | 0.00 | - |  | 0.00 | - |
|  | 2004 | 383 | 3 | 0.78 | 1.45 |  | 0.00 | - |  | 0.00 | - |  | 0.00 | - |  | 0.00 | - |  | 0.00 | - |
|  | 2005 | 330 | 3 | 0.28 | 0.83 |  | 0.00 | - |  | 0.00 | - |  | 0.00 | - |  | 0.00 | - |  | 0.00 | - |
| Haute-Normandie | 2001 | 189 | 1 | 0.00 | - |  | 0.00 | - |  | 0.00 | - |  | 0.00 | - |  | 0.00 | - |  | 0.00 | - |
|  | 2004 | 221 | 1 | 1.36 | - |  | 0.00 | - |  | 0.00 | - |  | 0.00 | - |  | 0.00 | - |  | 0.00 | - |
|  | 2005 | 278 | 1 | 0.36 | - |  | 0.00 | - |  | 0.00 | - |  | 0.00 | - |  | 0.00 | - |  | 0.00 | - |
| Ile-de-France | 2002 | 69 | 1 | 1.45 | - |  | 0.00 | - |  | 0.00 | - |  | 0.00 | - |  | 0.00 | - |  | 0.00 | - |
|  | 2003 | 124 | 2 | 0.00 | - |  | 0.00 | - |  | 0.00 | - |  | 0.00 | - |  | 0.00 | - |  | 0.00 | - |
|  | 2004 | 105 | 1 | 0.00 | - |  | 0.00 | - |  | 0.00 | - |  | 0.00 | - |  | 0.00 | - |  | 0.00 | - |
|  | 2005 | 161 | 1 | 0.00 | - |  | 0.00 | - |  | 0.00 | - |  | 0.00 | - |  | 0.00 | - |  | 0.00 | - |
| Languedoc-Roussillon | 2001 | 142 | 2 | 0.00 | - |  | 0.00 | - |  | 0.00 | - |  | 0.00 | - |  | 0.00 | - |  | 0.00 | - |
|  | 2002 | 112 | 1 | 0.00 | - |  | 0.00 | - |  | 0.00 | - |  | 0.00 | - |  | 0.00 | - |  | 0.00 | - |
|  | 2003 | 86 | 1 | 0.00 | - |  | 2.33 | - |  | 0.00 | - |  | 0.00 | - |  | 0.00 | - |  | 0.00 | - |
|  | 2004 | 128 | 1 | 0.00 | - |  | 0.00 | - |  | 0.00 | - |  | 0.00 | - |  | 0.00 | - |  | 0.00 | - |
|  | 2005 | 132 | 1 | 0.00 | - |  | 0.00 | - |  | 0.00 | - |  | 0.00 | - |  | 0.00 | - |  | 0.00 | - |
| Limousin | 2001 | 490 | 4 | 0.22 | 0.88 |  | 0.00 | - |  | 0.00 | - |  | 0.00 | - |  | 1.13 | 2.25 |  | 0.00 | - |
|  | 2002 | 298 | 3 | 0.70 | 2.11 |  | 0.00 | - |  | 0.00 | - |  | 0.00 | - |  | 0.00 | - |  | 0.00 | - |
|  | 2003 | 361 | 3 | 0.28 | 0.85 |  | 0.00 | - |  | 0.00 | - |  | 0.00 | - |  | 0.00 | - |  | 0.00 | - |
|  | 2004 | 321 | 3 | 0.90 | 1.77 |  | 0.00 | - |  | 0.00 | - |  | 0.00 | - |  | 0.00 | - |  | 0.00 | - |
|  | 2005 | 327 | 3 | 1.27 | 3.80 |  | 0.00 | - |  | 0.00 | - |  | 0.00 | - |  | 0.00 | - |  | 0.00 | - |
| Lorraine | 2002 | 276 | 4 | 0.00 | - |  | 0.00 | - |  | 0.00 | - |  | 0.00 | - |  | 0.00 | - |  | 0.00 | - |
|  | 2003 | 186 | 4 | 0.00 | - |  | 26.63 | 54.90 |  | 0.00 | - |  | 0.00 | - |  | 0.00 | - |  | 0.00 | - |
|  | 2005 | 452 | 4 | 0.00 | - |  | 0.00 | - |  | 0.00 | - |  | 0.00 | - |  | 0.00 | - |  | 0.00 | - |
| Midi-Pyrénées | 2001 | 1,502 | 10 | 0.15 | 0.79 |  | 0.00 | - |  | 0.00 | - |  | 0.00 | - |  | 0.08 | 0.77 |  | 0.00 | - |
|  | 2002 | 609 | 5 | 0.00 | - |  | 0.00 | - |  | 0.00 | - |  | 0.15 | 0.74 |  | 0.00 | - |  | 0.00 | - |
|  | 2003 | 1,054 | 5 | 0.00 | - |  | 0.00 | - |  | 0.00 | - |  | 0.00 | - |  | 0.00 | - |  | 0.00 | - |
|  | 2004 | 857 | 5 | 0.13 | 0.67 |  | 0.00 | - |  | 0.37 | 1.33 |  | 0.00 | - |  | 0.00 | - |  | 0.00 | - |
|  | 2005 | 789 | 5 | 0.13 | 0.65 |  | 0.00 | - |  | 0.00 | - |  | 0.00 | - |  | 0.00 | - |  | 0.00 | - |
| Pays de La Loire | 2001 | 983 | 7 | 0.30 | 0.73 |  | 0.00 | - |  | 0.00 | - |  | 0.00 | - |  | 0.00 | - |  | 0.00 | - |
|  | 2002 | 764 | 5 | 0.26 | 0.66 |  | 0.00 | - |  | 0.00 | - |  | 0.00 | - |  | 0.00 | - |  | 0.00 | - |
|  | 2003 | 707 | 5 | 0.23 | 1.15 |  | 0.00 | - |  | 0.00 | - |  | 0.00 | - |  | 0.00 | - |  | 0.00 | - |
|  | 2004 | 882 | 5 | 0.96 | 2.50 |  | 0.00 | - |  | 0.00 | - |  | 0.00 | - |  | 0.00 | - |  | 0.00 | - |
|  | 2005 | 1,059 | 5 | 0.66 | 2.76 |  | 0.00 | - |  | 0.00 | - |  | 0.00 | - |  | 0.44 | 1.66 |  | 0.00 | - |
| Picardie | 2005 | 88 | 1 | 0.00 | - |  | 0.00 | - |  | 0.00 | - |  | 0.00 | - |  | 0.00 | - |  | 0.00 | - |
| Poitou-Charentes | 2001 | 1,141 | 10 | 0.35 | 1.10 |  | 0.00 | - |  | 0.00 | - |  | 0.00 | - |  | 0.39 | 2.00 |  | 0.00 | - |
|  | 2002 | 762 | 5 | 0.71 | 2.15 |  | 0.00 | - |  | 0.00 | - |  | 0.36 | 1.23 |  | 0.24 | 1.19 |  | 0.00 | - |
|  | 2003 | 709 | 5 | 0.85 | 2.88 |  | 0.00 | - |  | 0.00 | - |  | 0.00 | - |  | 0.00 | - |  | 0.00 | - |
|  | 2004 | 778 | 5 | 0.65 | 1.15 |  | 0.11 | 0.57 |  | 0.00 | - |  | 0.00 | - |  | 0.16 | 0.79 |  | 0.00 | - |
|  | 2005 | 755 | 5 | 0.86 | 3.60 |  | 0.00 | - |  | 0.00 | - |  | 0.00 | - |  | 0.00 | - |  | 0.00 | - |
| Provence-Alpes-Côte d'Azur | 2001 | 103 | 1 | 0.00 | - |  | 0.00 | - |  | 0.00 | - |  | 0.00 | - |  | 0.00 | - |  | 0.00 | - |
|  | 2002 | 103 | 1 | 0.00 | - |  | 0.00 | - |  | 3.88 | - |  | 0.00 | - |  | 0.00 | - |  | 0.00 | - |
|  | 2003 | 362 | 1 | 0.00 | - |  | 0.28 | - |  | 0.83 | - |  | 0.00 | - |  | 0.00 | - |  | 0.00 | - |
|  | 2004 | 171 | 1 | 0.00 | - |  | 0.00 | - |  | 1.17 | - |  | 0.00 | - |  | 0.00 | - |  | 0.00 | - |
|  | 2005 | 147 | 1 | 0.00 | - |  | 0.00 | - |  | 0.68 | - |  | 0.00 | - |  | 0.00 | - |  | 0.00 | - |
| Rhône-Alpes | 2001 | 1,618 | 10 | 0.00 | - |  | 0.00 | - |  | 0.37 | 1.35 |  | 0.48 | 2.11 |  | 0.13 | 0.70 |  | 0.00 | - |
|  | 2002 | 787 | 5 | 0.00 | - |  | 0.00 | - |  | 3.43 | 4.55 |  | 0.64 | 3.18 |  | 0.25 | 1.27 |  | 0.00 | - |
|  | 2003 | 555 | 5 | 0.00 | - |  | 0.00 | - |  | 1.27 | 3.31 |  | 0.00 | - |  | 0.00 | - |  | 0.00 | - |
|  | 2004 | 1,446 | 5 | 0.24 | 0.88 |  | 0.00 | - |  | 0.43 | 1.52 |  | 0.00 | - |  | 0.35 | 0.72 |  | 0.00 | - |
|  | 2005 | 887 | 5 | 0.79 | 1.85 |  | 0.00 | - |  | 0.79 | 2.74 |  | 0.00 | - |  | 0.00 | - |  | 0.00 | - |
