# Supplementary material for: Changes in Parasitoid Communities Over Time and Space: A Historical Case Study of the Maize Pest Ostrinia nubilalis
Source: PLoS One. 2011 Sep 30;6(9):e25374. doi: 10.1371/journal.pone.0025374 (PMC3184128; doi:10.1371/journal.pone.0025374)
Supplement: Table S4 — Results of GLM analyses testing the “region”, “year” and the interaction “region x year” effects on mean parasitism rate (PR) per site and on % of infested sites. Values of Fisher (F) and Chi-square (χ2) are given for the gaussian and binomial models, respectively. df = degree of fredoom. (DOC) [file pone.0025374.s004.doc]

**Table S4** – Results of GLM analyses testing the “region”, “year” and the interaction “region x year” effects on mean parasitism rate (*PR*) per site and on % of infested sites. Values of Fisher (*F*) and Chi-square (*χ^2^*) are given for the gaussian and binomial models, respectively. *df* = degree of fredoom.

|  |  |  | **Statistical results (*F or χ^2^; df; p*-values)** | | | | |
| --- | --- | --- | --- | --- | --- | --- | --- |
| **Parasitoid** | **Indice** | **Model** | **Region** |  | **Year** |  | **Region x Year** |
| Tachinids | % infested sites | Binomial | 201.98; 18, 273; <0.001 |  | 0.17; 1, 272; 0.680 |  | 34.08; 17, 255; 0.010 |
|  | *PR* per site | Gaussian | 20.10; 18, 273; <0.001 |  | 0.25; 1, 272; 0.616 |  | 1.57; 17,255; 0.073 |
| Hymenopteran | % infested sites | Binomial | 14.01; 18, 273; <0.001 |  | <0.001; 1, 272; 0.976 |  | 5.47; 17, 255; 0.073 |
|  | *PR* per site | Gaussian | 2.32; 18, 273; 0.002 |  | 0.02; 1,272; 0.900 |  | 0.81; 17,255; 0.685 |
| Overall | % infested sites | Binomial | 132.96; 18, 273; <0.001 |  | 1.17; 1, 272; 0.280 |  | 44.18; 17, 255; 0.003 |
|  | *PR* per site | Gaussian | 9.92; 18, 273; <0.001 |  | 0.19; 1, 272; 0.665 |  | 1.37; 17,255; 0.151 |
