# Supplementary material for: Changes in Parasitoid Communities Over Time and Space: A Historical Case Study of the Maize Pest Ostrinia nubilalis
Source: PLoS One. 2011 Sep 30;6(9):e25374. doi: 10.1371/journal.pone.0025374 (PMC3184128; doi:10.1371/journal.pone.0025374)
Supplement: Table S5 — Results of GLM analyses testing the “region groups”, “year” and the interaction “region groups x year” effects on mean species richness (SR) per region, mean parasitism rate (PR) per site, Shannon and Weaver diversity Index (H′) per region and % infested sites. Values of Fisher (F) and Chi-square (χ2) are given for the gaussian and binomial/binomial negative models, respectively. df = degree of fredoom. (DOC) [file pone.0025374.s005.doc]

**Table S5** – Results of GLM analyses testing the “region groups”, “year” and the interaction “region groups x year” effects on mean species richness (*SR*) per region, mean parasitism rate (*PR*) per site, Shannon and Weaver diversity Index (*H'*) per region and % infested sites. Values of Fisher (*F*) and Chi-square (*χ^2^*) are given for the gaussian and binomial/binomial negative models, respectively. *df* = degree of fredoom.

|  |  |  | **Statistical results (*F* or *χ^2^, df, p*-values)** | | | | |
| --- | --- | --- | --- | --- | --- | --- | --- |
| **Parasitoid** | **Indice** | **Model** | **Region groups** |  | **Year** |  | **Region groups x Year** |
| Tachinids | % infested sites | Binomial | 18.94; 2, 289; <0.001 |  | 0.86; 1, 288; 0.350 |  | 0.27; 2, 286; 0.870 |
|  | *SR* per region | Binomial negative | 16.10; 2, 79; <0.001 |  | 0.002; 1, 78; 0.964 |  | 0.50; 2, 76; 0.777 |
|  | *PR* per site | Gaussian | 17.07; 2, 289; < 0.001 |  | 1.59; 1, 288; 0.209 |  | 0.40; 2, 286; 0.673 |
|  | *H'* per region | Gaussian | 2.45; 2, 26; 0.109 |  | 1.24; 1, 25; 0.276 |  | 0.16; 2, 23; 0.855 |
| Hymenopteran | % infested sites | Binomial | 16.62; 2, 289; <0.001 |  | 0.24; 1, 288; 0.620 |  | 4.95; 2, 286; 0.080 |
|  | *SR* per region | Binomial negative | 16.00; 2, 79; <0.001 |  | 0.51; 1, 78; 0.476 |  | 0.93; 2, 76; 0.627 |
|  | *PR* per site | Gaussian | 7.19; 2, 289; <0.001 |  | 0.06; 1, 288; 0.808 |  | 1.18; 2, 286; 0.309 |
|  | *H'* per region | Gaussian | 0.24; 2, 16; 0.787 |  | 0.03; 1, 15; 0.866 |  | 0.21; 2, 13; 0.810 |
| Overall | % infested sites | Binomial | 22.02; 2, 289; <0.001 |  | 0.08; 1, 288; 0.770 |  | 9.80; 2, 286; 0.010 |
|  | *SR* per region | Binomial negative | 26.21; 2, 79; <0.001 |  | 0.21; 1, 78; 0.646 |  | 0.72; 2, 76; 0.697 |
|  | *PR* per site | Gaussian | 20.42; 2, 289; <0.001 |  | 1.18; 1, 288; 0.278 |  | 1.37; 2, 286; 0.256 |
|  | *H'* per region | Gaussian | 3.65; 2, 45; 0.034 |  | 0.289; 1, 44; 0.594 |  | 0.25; 2, 42; 0.779 |
