# Supplementary material for: Changes in Parasitoid Communities Over Time and Space: A Historical Case Study of the Maize Pest Ostrinia nubilalis
Source: PLoS One. 2011 Sep 30;6(9):e25374. doi: 10.1371/journal.pone.0025374 (PMC3184128; doi:10.1371/journal.pone.0025374)
Supplement: Table S6 — Parasitism rates (in %) overall tachinids, overall hymenopteran and overall parasitoids infesting O. nubilalis and O. scapulalis. References: A = Thompson & Parker (1928), B = Paillot (1928), C = Parker et al. (1929), D = Pélissié et al. (2010), E = this study. Sd = standard deviation. * Not given but probably several thousands, ** Not given but probably several. (DOC) [file pone.0025374.s006.doc]

**Table S6** – Parasitism rates (in %) overall tachinids, overall hymenopteran and overall parasitoids infesting *O. nubilalis* and *O. scapulalis*. References: A = Thompson & Parker (1928), B = Paillot (1928), C = Parker et al. (1929), D = Pélissié et al. (2010), E = this study. Sd = standard deviation. * Not given but probably several thousands, ** Not given but probably several.

|  |  |  |  |  | **Overall**  **parasitism** | | |  | **Tachinids** | | |  | **Hymenopteran** | | |  |
| --- | --- | --- | --- | --- | --- | --- | --- | --- | --- | --- | --- | --- | --- | --- | --- | --- |
| **Host species** | **Region** | **Period** | **N larvae** | **N sites** | **Mean** | **Sd** | **Max** |  | **Mean** | **Sd** | **Max** |  | **Mean** | **Sd** | **Max** | **Reference** |
| *O. nubilalis* | Alsace | 1925 | 500 | ** | 7.80 | - | - |  | 0.00 | - | - |  | 7.80 | - | - | A |
|  |  | 2001 to 2005 | 5,483 | 28 | 1.22 | 1.42 | 10.53 |  | 0.78 | 1.23 | 10.53 |  | 0.42 | 0.73 | 8.76 | E |
|  | Aquitaine | 1921 to 1925 | * | ** | 16.37 | - | 22.70 |  | 5.70 | - | - |  | 10.67 | - | 27.90 | A |
|  |  | 1926 to 1928 | * | ** | 8.93 | 5.12 | - |  | 6.33 | 5.29 | - |  | 2.59 | 0.17 | - | C |
|  |  | 2001 to 2005 | 3,886 | 25 | 7.75 | 2.09 | 16.24 |  | 6.11 | 2.03 | 15.23 |  | 0.92 | 0.37 | 3.45 | E |
|  | Auvergne | 2001 to 2005 | 1,952 | 10 | 0.15 | 0.31 | 1.23 |  | 0.00 | - | - |  | 0.15 | 0.31 | 1.23 | E |
|  | Bourgogne | 1927 | 102 | 3 | ? | - | - |  | - | - | - |  | 7.84 | - | 14.71 | C |
|  |  | 2001 to 2005 | 1,930 | 14 | 2.31 | 2.03 | 9.29 |  | 0.36 | 0.33 | 1.43 |  | 1.69 | 1.58 | 9.29 | E |
|  | Bretagne | 2001 to 2005 | 1,645 | 12 | 0.38 | 0.85 | 2.97 |  | 0.00 | - | - |  | 0.26 | 0.57 | 2.97 | E |
|  | Centre | 2001 to 2005 | 1,476 | 14 | 1.74 | 3.47 | 13.16 |  | 1.18 | 2.35 | 8.33 |  | 0.51 | 1.01 | 4.17 | E |
|  | Champagne-Ardenne | 2001 to 2005 | 1,271 | 11 | 1.21 | 1.15 | 6.06 |  | 0.08 | 0.17 | 0.76 |  | 1.02 | 1.11 | 5.30 | E |
|  | Franche-Comté | 1924 to1925 | > 1,250 | ** | 8.10 | - | - |  | 0.45 | - | - |  | 5.55 | - | 5.70 | A |
|  |  | 1927 to 1928 | 163 | 2 | ? | - | - |  | - | - | - |  | 11.19 | 5.61 | 15.63 | C |
|  |  | 2001 to 2005 | 969 | 9 | 0.44 | 0.54 | 2.17 |  | 0.00 | - | - |  | 0.35 | 0.40 | 1.45 | E |
|  | Haute-Normandie | 2001 to 2005 | 688 | 3 | 2.02 | 1.75 | 3.17 |  | 1.44 | 1.30 | 2.52 |  | 0.57 | 0.70 | 1.36 | E |
|  | Ile-de-France | 2002 to 2005 | 459 | 5 | 0.98 | 1.21 | 2.48 |  | 0.62 | 1.24 | 2.48 |  | 0.36 | 0.72 | 1.45 | E |
|  | Languedoc-Roussillon | 2001 to 2005 | 600 | 6 | 2.02 | 1.53 | 3.49 |  | 1.08 | 1.13 | 2.68 |  | 0.47 | 1.04 | 2.33 | E |
|  | Limousin | 2001 to 2005 | 1,797 | 16 | 17.11 | 10.08 | 47.37 |  | 14.78 | 9.35 | 45.26 |  | 0.90 | 0.44 | 3.80 | E |
|  | Lorraine | 2002 to 2005 | 914 | 12 | 10.73 | 18.39 | 70.97 |  | 1.69 | 2.73 | 19.35 |  | 8.88 | 15.37 | 54.90 | E |
|  | Midi-Pyrénées | 2001 to 2005 | 3,757 | 30 | 5.38 | 0.95 | 13.48 |  | 4.58 | 0.92 | 12.77 |  | 0.20 | 0.19 | 1.33 | E |
|  | Pays de La Loire | 2001 to 2005 | 4,395 | 27 | 3.78 | 1.42 | 14.36 |  | 3.00 | 1.07 | 9.39 |  | 0.57 | 0.42 | 2.76 | E |
|  | Picardie | 2005 | 88 | 1 | 0.00 | - | - |  | 0.00 | - | - |  | 0.00 | - | - | E |
|  | Poitou-Charentes | 2001 to 2005 | 4,145 | 30 | 7.10 | 1.42 | 14.89 |  | 5.96 | 1.48 | 12.77 |  | 0.94 | 0.22 | 3.60 | E |
|  | Provence-Alpes-Côte d'Azur | 1921 to 1925 | > 3,333 | ** | 21.66 | - | 45.35 |  | 15.57 | - | - |  | 5.06 | - | 18.80 | A |
|  |  | 1926 to 1928 | * | ** | 12.46 | 8.38 | - |  | 7.62 | 4.01 | - |  | 6.16 | 8.31 | - | C |
|  |  | 2001 to 2005 | 886 | 5 | 4.99 | 3.69 | 8.84 |  | 2.71 | 2.41 | 6.12 |  | 1.37 | 1.48 | 3.88 | E |
|  | Rhône-Alpes | 1925 | 770 | 1 | 17.90 | - | - |  | 8.90 | - | - |  | 9.00 | - | - | A |
|  |  | 1926 to 1928 | * | ** | 4.85 | 4.88 | - |  | 4.50 | 4.81 | - |  | 0.35 | 0.07 | - | C |
|  |  | 1927 to 1928 | 336 | 4 | 9.95 | - | 15.09 |  | 9.48 | - | 14.15 |  | 6.32 | 7.36 | 14.58 | C |
|  |  | 2001 to 2005 | 5,293 | 30 | 4.43 | 2.48 | 12.10 |  | 1.83 | 1.70 | 7.01 |  | 1.83 | 1.41 | 4.55 | E |
| *O. scapulalis* | Alsace | 1925 | >200 | ** | 17.50 | - | - |  | 0.00 | - | - |  | 17.50 | - | - | A |
|  | Auvergne | 1927 | * | ** | 24.80 | - | - |  | 1.20 | - | - |  | 23.60 | - | - | C |
|  | Centre | 2002 | 114 | 1 | 22.81 | - | 19.46 |  | 0.00 | - | - |  | 22.81 | - | 19.46 | D |
|  | Franche-Comté | 1924 to 1925 | > 400 | ** | 8.25 | - | 14.70 |  | 0.25 | - | - |  | 8.00 | - | 9.00 | A |
|  | Ile-de-France | 1922 to 1925 | * | ** | 32.55 | - | 66.70 |  | 0.00 | - | - |  | 32.55 | - | 63.20 | A |
|  |  | 1926 to 1928 | * | ** | 38.60 | 12.85 | - |  | 0.00 | - | - |  | 38.61 | 12.85 | - | C |
|  |  | 2002 | 149 | 1 | 19.46 | - | 19.46 |  | 0.00 | - | - |  | 19.46 | - | 19.46 | D |
|  | Lorraine | 1926 | * | ** | 2.80 | - | - |  | 0.00 | - | - |  | 2.80 | - | - | C |
|  | Nord-Pas de Calais | 1922 to 1925 | * | ** | 38.85 | - | 58.90 |  | 0.00 | - | - |  | 38.85 | - | 42.60 | A |
|  |  | 1926 to 1928 | * | ** | 22.37 | 2.14 | - |  | 0.00 | - | - |  | 20.08 | 5.57 | - | C |
|  |  | 2002 | 199 | 2 | 15.16 | - | 29.29 |  | 0.51 | - | 1.01 |  | 14.65 | - | 29.29 | D |
|  | Pays de La Loire | 1925 | 1,000 | ** | 10.50 | - | - |  | 0.00 | - | - |  | 10.50 | - | - | A |
|  |  | 1926 to 1928 | * | ** | 34.17 | 2.30 | - |  | 0.00 | - | - |  | 34.17 | 2.30 | - | C |
|  | Picardie | 2002 | 265 | 3 | 13.93 | - | 34.78 |  | 0.00 | - | - |  | 13.93 | - | 34.78 | D |
