# Supplementary material for: Changes in Parasitoid Communities Over Time and Space: A Historical Case Study of the Maize Pest Ostrinia nubilalis
Source: PLoS One. 2011 Sep 30;6(9):e25374. doi: 10.1371/journal.pone.0025374 (PMC3184128; doi:10.1371/journal.pone.0025374)
Supplement: Table S8 — Parasitism rates (in %) of the hymenopteran Braconidae infesting O. nubilalis and O. scapulalis. Parasitism rates of Eulophidae were negligible, and thus not given. References: A = Thompson & Parker (1928), B = Paillot (1928), C = Parker et al. (1929), D = Pélissié et al. (2010), E = this study. Sd = standard deviation. * Not given but probably several thousands, ** Not given but probably several. (DOC) [file pone.0025374.s008.doc]

**Table S8** – Parasitism rates (in %) of the hymenopteran Braconidae infesting *O. nubilalis* and *O. scapulalis*. Parasitism rates of Eulophidae were negligible*,* and thus not given. References: A = Thompson & Parker (1928), B = Paillot (1928), C = Parker et al. (1929), D = Pélissié et al. (2010), E = this study. Sd = standard deviation. * Not given but probably several thousands, ** Not given but probably several.

|  |  |  |  |  | ***Apanteles thompsoni*** | | |  | ***Bracon***  ***brevicornis*** | | |  | ***Macrocentrus cingulum*** | | |  | ***Microgaster messoria*** | | |  |
| --- | --- | --- | --- | --- | --- | --- | --- | --- | --- | --- | --- | --- | --- | --- | --- | --- | --- | --- | --- | --- |
| **Host species** | **Region** | **Period** | **N larvae** | **N sites** | **Mean** | **Sd** | **Max** |  | **Mean** | **Sd** | **Max** |  | **Mean** | **Sd** | **Max** |  | **Mean** | **Sd** | **Max** | **Reference** |
| *O. nubilalis* | Alsace | 1925 | 500 | ** | 0.00 | - | - |  | 0.00 | - | - |  | 0.00 | - | - |  | 1.40 | - | - | A |
|  |  | 2001 to 2005 | 5,483 | 28 | 0.00 | - | - |  | 0.34 | 0.77 | 8.76 |  | 0.00 | - | - |  | 0.00 | - | - | E |
|  | Aquitaine | 1921 to 1925 | * | ** | 0.00 | - | - |  | 0.00 | - | - |  | 0.00 | - | - |  | 0.00 | - | - | A |
|  |  | 1926 to 1928 | * | ** | 0.00 | - | - |  | 0.00 | - | - |  | 0.00 | - | - |  | 0.28 | 0.36 | - | C |
|  |  | 2001 to 2005 | 3,886 | 25 | 0.00 | - | - |  | 0.00 | - | - |  | 0.00 | - | - |  | 0.02 | 0.05 | 0.59 | E |
|  | Auvergne | 2001 to 2005 | 1,952 | 10 | 0.00 | - | - |  | 0.00 | - | - |  | 0.00 | - | - |  | 0.00 | - | - | E |
|  | Bourgogne | 1927 | 102 | 3 | - | - | - |  | - | - | - |  | - | - | - |  | - | - | - | C |
|  |  | 2001 to 2005 | 1,930 | 14 | 0.00 | - | - |  | 0.00 | - | - |  | 0.00 | - | - |  | 0.00 | - | - | E |
|  | Bretagne | 2001 to 2005 | 1,645 | 12 | 0.00 | - | - |  | 0.00 | - | - |  | 0.00 | - | - |  | 0.00 | - | - | E |
|  | Centre | 2001 to 2005 | 1,476 | 14 | 0.00 | - | - |  | 0.00 | - | - |  | 0.00 | - | - |  | 0.00 | - | - | E |
|  | Champagne-Ardenne | 2001 to 2005 | 1,271 | 11 | 0.00 | - | - |  | 0.00 | - | - |  | 0.00 | - | - |  | 0.00 | - | - | E |
|  | Franche-Comté | 1924 to 1925 | > 1,250 | ** | 0.08 | - | 0.15 |  | 0.00 | - | - |  | 0.00 | - | - |  | 0.00 | - | - | A |
|  |  | 1927 to 1928 | 163 | 2 | - | - | - |  | - | - | - |  | - | - | - |  | - | - | - | C |
|  |  | 2001 to 2005 | 969 | 9 | 0.00 | - | - |  | 0.00 | - | - |  | 0.00 | - | - |  | 0.00 | - | - | E |
|  | Haute-Normandie | 2001 to 2005 | 688 | 3 | 0.00 | - | - |  | 0.00 | - | - |  | 0.00 | - | - |  | 0.00 | - | - | E |
|  | Ile-de-France | 2002 to 2005 | 459 | 5 | 0.00 | - | - |  | 0.00 | - | - |  | 0.00 | - | - |  | 0.00 | - | - | E |
|  | Languedoc-Roussillon | 2001 to 2005 | 600 | 6 | 0.00 | - | - |  | 0.47 | 1.04 | 2.33 |  | 0.00 | - | - |  | 0.00 | - | - | E |
|  | Limousin | 2001 to 2005 | 1,797 | 16 | 0.00 | - | - |  | 0.00 | - | - |  | 0.00 | - | - |  | 0.00 | - | - | E |
|  | Lorraine | 2002 to 2005 | 914 | 12 | 0.00 | - | - |  | 8.88 | 15.37 | 54.90 |  | 0.00 | - | - |  | 0.00 | - | - | E |
|  | Midi-Pyrénées | 2001 to 2005 | 3,757 | 30 | 0.00 | - | - |  | 0.00 | - | - |  | 0.00 | - | - |  | 0.03 | 0.07 | 0.74 | E |
|  | Pays de La Loire | 2001 to 2005 | 4,395 | 27 | 0.00 | - | - |  | 0.00 | - | - |  | 0.00 | - | - |  | 0.00 | - | - | E |
|  | Picardie | 2005 | 88 | 1 | 0.00 | - | - |  | 0.00 | - | - |  | 0.00 | - | - |  | 0.00 | - | - | E |
|  | Poitou-Charentes | 2001 to 2005 | 4,145 | 30 | 0.00 | - | - |  | 0.02 | 0.05 | 0.57 |  | 0.00 | - | - |  | 0.07 | 0.16 | 1.23 | E |
|  | Provence-Alpes-Côte d'Azur | 1921 to 1925 | > 3,333 | ** | 0.00 | - | - |  | 0.03 | - | 0.23 |  | 0.00 | - | - |  | 0.00 | - | - | A |
|  |  | 1926 to 1928 | * | ** | 1.32 | 2.95 | - |  | 0.00 | - | - |  | 0.00 | - | - |  | 0.00 | - | - | C |
|  |  | 2001 to 2005 | 886 | 5 | 0.00 | - | - |  | 0.06 | 0.12 | 0.28 |  | 0.00 | - | - |  | 0.00 | - | - | E |
|  | Rhône-Alpes | 1925 | 770 | 1 | 0.00 | - | - |  | 0.60 | - | - |  | 0.00 | - | - |  | 0.00 | - | - | A |
|  |  | 1926 to 1928 | * | ** | 0.00 | - | - |  | 0.10 | 0.14 | - |  | 0.00 | - | - |  | 0.00 | - | - | C |
|  |  | 1927 to 1928 | 336 | 4 | 0.00 | - | - |  | 0.00 | - | - |  | 0.00 | - | - |  | 0.00 | - | - | C |
|  |  | 2001 to 2005 | 5,293 | 30 | 0.00 | - | - |  | 0.00 | - | - |  | 0.00 | - | - |  | 0.22 | 0.31 | 3.18 | E |
| *O. scapulalis* | Alsace | 1925 | >200 | ** | 0.00 | - | - |  | 0.00 | - | - |  | 0.00 | - | - |  | 13.80 | - | - | A |
|  | Auvergne | 1927 | * | ** | 0.00 | - | - |  | 0.00 | - | - |  | 22.70 | - | - |  | 0.00 | - | - | C |
|  | Centre | 2002 | 114 | 1 | - | - | - |  | - | - | - |  | 22.81 | - | 19.46 |  | - | - | - | D |
|  | Franche-Comté | 1924 to 1925 | > 400 | ** | 1.10 | - | 1.80 |  | 0.00 | - | - |  | 0.00 | - | - |  | 1.35 | - | 2.70 | A |
|  | Ile-de-France | 1922 to 1925 | * | ** | 0.00 | - | - |  | 0.00 | - | - |  | 0.00 | - | - |  | 31.40 | - | 63.20 | A |
|  |  | 1926 to 1928 | * | ** | 0.00 | - | - |  | 0.00 | - | - |  | 0.67 | 1.15 | - |  | 36.80 | 13.80 | - | C |
|  |  | 2002 | 149 | 1 | - | - | - |  | - | - | - |  | 19.46 | - | 19.46 |  | - | - | - | D |
|  | Lorraine | 1926 | * | ** | 0.00 | - | - |  | 0.00 | - | - |  | 0.00 | - | - |  | 2.20 | - | - | C |
|  | Nord-Pas de Calais | 1922 to 1925 | * | ** | 22.90 | - | 42.60 |  | 0.00 | - | - |  | 0.00 | - | - |  | 15.00 | - | 18.70 | A |
|  |  | 1926 to 1928 | * | ** | 1.90 | 1.68 | - |  | 0.00 | - | - |  | 0.00 | - | - |  | 17.57 | 4.17 | - | C |
|  |  | 2002 | 199 | 2 | - | - | - |  | - | - | - |  | 14.65 | - | 29.29 |  | - | - | - | D |
|  | Pays de La Loire | 1925 | 1,000 | ** | 0.00 | - | - |  | 0.00 | - | - |  | 8.90 | - | - |  | 0.40 | - | - | A |
|  |  | 1926 to 1928 | * | ** | 0.00 | - | - |  | 0.00 | - | - |  | 33.67 | 2.37 | - |  | 0.13 | 0.06 | - | C |
|  | Picardie | 2002 | 265 | 3 | - | - | - |  | - | - | - |  | 13.93 | - | 34.78 |  | - | - | - | D |
