# Supplementary material for: Changes in Parasitoid Communities Over Time and Space: A Historical Case Study of the Maize Pest Ostrinia nubilalis
Source: PLoS One. 2011 Sep 30;6(9):e25374. doi: 10.1371/journal.pone.0025374 (PMC3184128; doi:10.1371/journal.pone.0025374)
Supplement: Table S9 — Parasitism rates (in %) of the hymenopteran Ichneumonidae infesting O. nubilalis and O. scapulalis. Parasitism rates of Campoplex lugubrinus, Campoplex rothi, Exeristes roborator, Pristomerus vulnerator and Theronia atalantae were negligible and are therefore not given. References: A = Thompson & Parker (1928), B = Paillot (1928), C = Parker et al. (1929), D = Pélissié et al. (2010), E = this study. Sd = standard deviation. * Not given but probably several thousands, ** Not given but probably several. (DOC) [file pone.0025374.s009.doc]

**Table S9** – Parasitism rates (in %) of the hymenopteran Ichneumonidae infesting *O. nubilalis* and *O. scapulalis*. Parasitism rates of *Campoplex lugubrinus*, *Campoplex rothi*, *Exeristes roborator*, *Pristomerus vulnerator* and *Theronia atalantae* were negligible and are therefore not given. References: A = Thompson & Parker (1928), B = Paillot (1928), C = Parker et al. (1929), D = Pélissié et al. (2010), E = this study. Sd = standard deviation. * Not given but probably several thousands, ** Not given but probably several.

|  |  |  |  |  | ***Diadegma fenestrale*** | | |  | ***Eriborus***  ***terebrans*** | | |  | ***Phaogenes planifrons*** | | |  | ***Sinophorus turionum*** | | |  |
| --- | --- | --- | --- | --- | --- | --- | --- | --- | --- | --- | --- | --- | --- | --- | --- | --- | --- | --- | --- | --- |
| **Host species** | **Region** | **Period** | **N larvae** | **N sites** | **Mean** | **Sd** | **Max** |  | **Mean** | **Sd** | **Max** |  | **Mean** | **Sd** | **Max** |  | **Mean** | **Sd** | **Max** | **Reference** |
| *O. nubilalis* | Alsace | 1925 | 500 | ** | 0.00 | - | - |  | 0.00 | - | - |  | 0.00 | - | - |  | 6.40 | - | - | A |
|  |  | 2001 to 2005 | 5,483 | 28 | 0.01 | 0.03 | 0.43 |  | 0.00 | - | - |  | 0.00 | - | - |  | 0.04 | 0.08 | 0.44 | E |
|  | Aquitaine | 1921 to 1925 | * | ** | 0.00 | - | - |  | 0.80 | - | 1.70 |  | 0.00 | - | - |  | 9.87 | - | 27.90 | A |
|  |  | 1926 to 1928 | * | ** | 0.00 | - | - |  | 0.01 | 0.01 | - |  | 0.00 | - | - |  | 2.30 | 0.52 | - | C |
|  |  | 2001 to 2005 | 3,886 | 25 | 0.05 | 0.07 | 0.68 |  | 0.36 | 0.39 | 2.42 |  | 0.00 | - | - |  | 0.46 | 0.37 | 3.45 | E |
|  | Auvergne | 2001 to 2005 | 1,952 | 10 | 0.00 | - | - |  | 0.15 | 0.31 | 1.23 |  | 0.00 | - | - |  | 0.00 | - | - | E |
|  | Bourgogne | 1927 | 102 | 3 | - | - | - |  | - | - | - |  | - | - | - |  | 7.84 | - | 14.71 | C |
|  |  | 2001 to 2005 | 1,930 | 14 | 0.00 | - | - |  | 0.00 | - | - |  | 0.00 | - | - |  | 1.69 | 1.58 | 9.29 | E |
|  | Bretagne | 2001 to 2005 | 1,645 | 12 | 0.00 | - | - |  | 0.00 | - | - |  | 0.00 | - | - |  | 0.26 | 0.57 | 2.97 | E |
|  | Centre | 2001 to 2005 | 1,476 | 14 | 0.22 | 0.44 | 2.70 |  | 0.00 | - | - |  | 0.00 | - | - |  | 0.28 | 0.57 | 4.17 | E |
|  | Champagne-Ardenne | 2001 to 2005 | 1,271 | 11 | 0.00 | - | - |  | 0.00 | - | - |  | 0.00 | - | - |  | 1.02 | 1.11 | 5.30 | E |
|  | Franche-Comté | 1924 to 1925 | > 1,250 | ** | 0.00 | - | - |  | 0.00 | - | - |  | 0.12 | - | 0.25 |  | 5.35 | - | 5.70 | A |
|  |  | 1927 to 1928 | 163 | 2 | - | - | - |  | - | - | - |  | - | - | - |  | 11.19 | 5.61 | 15.63 | C |
|  |  | 2001 to 2005 | 969 | 9 | 0.00 | - | - |  | 0.00 | - | - |  | 0.00 | - | - |  | 0.35 | 0.40 | 1.45 | E |
|  | Haute-Normandie | 2001 to 2005 | 688 | 3 | 0.00 | - | - |  | 0.00 | - | - |  | 0.00 | - | - |  | 0.57 | 0.70 | 1.36 | E |
|  | Ile-de-France | 2002 to 2005 | 459 | 5 | 0.00 | - | - |  | 0.00 | - | - |  | 0.00 | - | - |  | 0.36 | 0.72 | 1.45 | E |
|  | Languedoc-Roussillon | 2001 to 2005 | 600 | 6 | 0.00 | - | - |  | 0.00 | - | - |  | 0.00 | - | - |  | 0.00 | - | - | E |
|  | Limousin | 2001 to 2005 | 1,797 | 16 | 0.23 | 0.51 | 2.25 |  | 0.00 | - | - |  | 0.00 | - | - |  | 0.68 | 0.44 | 3.80 | E |
|  | Lorraine | 2002 to 2005 | 914 | 12 | 0.00 | - | - |  | 0.00 | - | - |  | 0.00 | - | - |  | 0.00 | - | - | E |
|  | Midi-Pyrénées | 2001 to 2005 | 3,757 | 30 | 0.02 | 0.03 | 0.77 |  | 0.07 | 0.17 | 1.33 |  | 0.00 | - | - |  | 0.08 | 0.08 | 0.79 | E |
|  | Pays de La Loire | 2001 to 2005 | 4,395 | 27 | 0.09 | 0.20 | 1.66 |  | 0.00 | - | - |  | 0.00 | - | - |  | 0.48 | 0.32 | 2.76 | E |
|  | Picardie | 2005 | 88 | 1 | 0.00 | - | - |  | 0.00 | - | - |  | 0.00 | - | - |  | 0.00 | - | - | E |
|  | Poitou-Charentes | 2001 to 2005 | 4,145 | 30 | 0.16 | 0.17 | 2.00 |  | 0.00 | - | - |  | 0.00 | - | - |  | 0.68 | 0.21 | 3.60 | E |
|  | Provence-Alpes-Côte d'Azur | 1921 to 1925 | > 3,333 | ** | 0.00 | - | - |  | 4.60 | - | 18.80 |  | 0.15 | - | 0.40 |  | 0.00 | - | - | A |
|  |  | 1926 to 1928 | * | ** | 0.00 | - | - |  | 4.70 | 8.80 | - |  | 0.00 | - | - |  | 0.00 | - | - | C |
|  |  | 2001 to 2005 | 886 | 5 | 0.00 | - | - |  | 1.31 | 1.50 | 3.88 |  | 0.00 | - | - |  | 0.00 | - | - | E |
|  | Rhône-Alpes | 1925 | 770 | 1 | 0.00 | - | - |  | 2.10 | - | - |  | 0.00 | - | - |  | 6.20 | - | - | A |
|  |  | 1926 to 1928 | * | ** | 0.00 | - | - |  | 0.25 | 0.07 | - |  | 0.00 | - | - |  | 0.00 | - | - | C |
|  |  | 1927 to 1928 | 336 | 4 | 0.00 | - | - |  | 0.00 | - | - |  | 0.00 | - | - |  | 6.32 | 7.36 | 14.58 | C |
|  |  | 2001 to 2005 | 5,293 | 30 | 0.15 | 0.15 | 1.27 |  | 1.26 | 1.27 | 4.55 |  | 0.00 | - | - |  | 0.21 | 0.34 | 1.85 | E |
| *O. scapulalis* | Alsace | 1925 | >200 | ** | 0.00 | - | - |  | 0.00 | - | - |  | 0.00 | - | - |  | 3.70 | - | - | A |
|  | Auvergne | 1927 | * | ** | 0.00 | - | - |  | 0.00 | - | - |  | 0.00 | - | - |  | 0.90 | - | - | C |
|  | Centre | 2002 | 114 | 1 | - | - | - |  | - | - | - |  | - | - | - |  | - | - | - | D |
|  | Franche-Comté | 1924 to 1925 | > 400 | ** | 0.00 | - | - |  | 0.00 | - | - |  | 0.35 | - | 0.70 |  | 5.20 | - | 9.00 | A |
|  | Ile-de-France | 1922 to 1925 | * | ** | 0.00 | - | - |  | 0.00 | - | - |  | 0.00 | - | - |  | 1.15 | - | 3.50 | A |
|  |  | 1926 to 1928 | * | ** | 0.00 | - | - |  | 0.00 | - | - |  | 0.00 | - | - |  | 1.00 | 0.70 | - | C |
|  |  | 2002 | 149 | 1 | - | - | - |  | - | - | - |  | - | - | - |  | - | - | - | D |
|  | Lorraine | 1926 | * | ** | 0.00 | - | - |  | 0.00 | - | - |  | 0.00 | - | - |  | 0.60 | - | - | C |
|  | Nord-Pas de Calais | 1922 to 1925 | * | ** | 0.00 | - | - |  | 0.00 | - | - |  | 0.00 | - | - |  | 0.95 | - | 1.90 | A |
|  |  | 1926 to 1928 | * | ** | 0.00 | - | - |  | 0.00 | - | - |  | 0.00 | - | - |  | 0.33 | 0.06 | - | C |
|  |  | 2002 | 199 | 2 | - | - | - |  | - | - | - |  | - | - | - |  | - | - | - | D |
|  | Pays de La Loire | 1925 | 1,000 | ** | 0.00 | - | - |  | 0.00 | - | - |  | 0.00 | - | - |  | 1.20 | - | - | A |
|  |  | 1926 to 1928 | * | ** | 0.00 | - | - |  | 0.00 | - | - |  | 0.00 | - | - |  | 0.37 | 0.25 | - | C |
|  | Picardie | 2002 | 265 | 3 | - | - | - |  | - | - | - |  | - | - | - |  | - | - | - | D |
